# Longitudinal Profiles of Cultural Identity Processes and Associations with Psychosocial Outcomes Among Adolescents Participating in the *Identity Project* in Italy

**DOI:** 10.1007/s10964-024-02022-6

**Published:** 2024-05-29

**Authors:** Chiara Ceccon, Ughetta Moscardino, Gianmarco Altoè, Francesca Lionetti, Adriana J. Umaña-Taylor

**Affiliations:** 1 University of Padova, Padua, Italy; 2 University of Chieti-D’Annunzio, Chieti, Italy; 3 Harvard Graduate School of Education, Cambridge, USA

**Keywords:** Identity Project, Intervention, Ethnic-racial identity, Longitudinal latent profile analysis, Adolescence, School

## Abstract

Cultural identity formation is a complex developmental task that influences adolescents’ adjustment. However, less is known about individual variations in trajectories of cultural identity processes and how they relate to youth psychosocial outcomes. Using a person-centered approach, this study investigated patterns of change over a year in cultural identity exploration and resolution, respectively, among ethnically diverse adolescents in Italy. The sample included 173 high school students (*M*_age_ = 15 yrs, *SD* = 0.62, range = 14–17; 58.4% female; 26% immigrant background) who had participated in the *Identity Project*, a school-based intervention targeting ethnic-racial identity development. Longitudinal latent profile analysis revealed only one profile of change for exploration, whereas four unique profiles for resolution emerged (“stable low,” “stable average,” “increase low-to-average,” “increase high-to-higher”). Overall, youth in the resolution-increase profiles reported the best outcomes. The findings highlight the heterogeneity of adolescents’ resolution trajectories and the benefits of an increased sense of clarity concerning one’s cultural identity for positive psychosocial functioning.

## Introduction

Ethnic-racial identity is a multidimensional construct encompassing the knowledge, beliefs, and attitudes individuals have toward their ethnoracial group membership(s), as well as the processes through which these aspects develop (Umaña‐Taylor et al. (2014)). Though this construct emerged initially in the U.S. and is grounded in the sociocultural and historical context of that nation, it has become increasingly relevant also in Europe given the rising number of immigrant and refugee arrivals in the past decade (Syed et al., 2018). In adolescence, exploring and gaining a sense of clarity regarding one’s own ethnic-racial identity is an important developmental task, especially in globalized societies where minoritized and majority youth are called upon to define their identity and learn how to approach cultural diversity (Schwarzenthal, 2022). Previous research indicates that a positive ethnic-racial identity is linked to better outcomes in terms of socio-emotional well-being, interpersonal relationships, and academic functioning (Umaña-Taylor & Rivas-Drake, 2021). However, most studies conducted to date have used variable-centered approaches to evaluate how ethnic-racial identity develops over time, impeding the identification of subgroups of adolescents whose trajectories may differentially be associated with psychosocial adjustment (Wantchekon & Umaña-Taylor, 2021). This study used a person-centered approach to identify profiles of cultural identity exploration and resolution trajectories over a year in adolescents who participated in a school-based intervention targeting ethnic-racial identity formation (i.e., the *Identity Project;* Umaña-Taylor & Douglass, 2017). Associations of the emerging profiles with individual (i.e., immigrant background) and contextual characteristics (i.e., family ethnic socialization) as well as their links to adolescents’ psychosocial adjustment were also examined. Understanding the various patterns of trajectories of exploration and resolution in the school environment is an essential step toward gaining insight into the development of these pivotal processes among specific subgroups of youth who may differentially benefit from interventions aiming to promote positive adjustment in multicultural settings.

### Ethnic-Racial Identity and Psychosocial Adjustment in Adolescence

Identity formation is a key developmental task in adolescence, prompted by cognitive maturation and increases in social autonomy that allow individuals to reflect on and practically explore different sets of values, goals, and potential identities (Erikson, 1968). Gaining deeper awareness and understanding of various identity components through an exploration phase fosters a definition of and cohesion within one’s general identity, reducing role confusion, contributing to psychological well-being, and ultimately enabling youth to cultivate healthy relationships and nurture a positive self-concept (Marcia, 1980). The two differential processes of exploration and resolution have also been applied to the development of ethnic-racial identity (Umaña-Taylor et al., 2004). Exploration involves actively searching, observing, and reflecting upon one’s identity and heritage (e.g., by learning more about the history of one’s ethnoracial group and participating in relevant cultural events and traditions). This process is critical for ethnic-racial identity development because individuals can achieve a definition of this part of their identity only by discovering and getting to know more about their cultural backgrounds (Phinney, 1989). Engaging in exploration helps reaching resolution (also referred to as commitment), which involves understanding the role ethnic-racial identity plays in one’s life and general sense of self, and developing a sense of clarity about the meaning this part of one’s identity has for oneself (Umaña-Taylor et al., 2004). Resolution undergoes changes as youth become more active in their exploration of the culture, history, and traditions of their ethnoracial group during adolescence (Huang & Stormshak, 2011).

Ethnic-racial identity, including components of exploration and resolution, has been found to be a predictor of positive psychosocial adjustment in terms of global identity cohesion (Umaña-Taylor et al., 2018b), self-esteem (Smokowski et al., 2014), depressive symptoms (Brittian et al. 2015), academic achievement (Miller-Cotto & Byrnes, 2016), other group orientation (Phinney et al., 2007), and prosocial behavior (Streit et al., 2020). Despite empirical evidence suggesting that ethnic-racial identity process components are closely related, they have been shown to be differentially associated with adjustment (e.g., Yip et al., 2019) and may have distinct developmental pathways, uniquely contributing to various outcomes. For instance, exploration was positively associated with life satisfaction, whereas resolution was linked to self-esteem and psychological well-being among British-born adolescents of Turkish descent living in England (Cavdar et al., 2021). A longitudinal study conducted in the U.S. found that growth in exploration increased the risk of depressive symptoms, while resolution was not significantly associated with this outcome variable in Mexican-origin adolescent girls (Gonzales-Backen et al., 2016). In this study, exploration and resolution were investigated as related, yet distinct components of ethnic-racial identity to shed light on potential differences in their developmental trajectories and associations with psychosocial outcomes.

The available literature on adolescents’ ethnic-racial/cultural identity is largely based on cross-sectional data to identify configurations of exploration and resolution/commitment and their associations with psychosocial adjustment (e.g., Meca et al., 2023), while less is known about the developmental trajectories of these processes in adolescence. The few extant longitudinal studies have mostly used a variable-centered framework focusing on mean levels of identity scores and relating them to different outcomes (Jugert et al., 2020). For instance, a study on ethnic-racial identity (exploration and commitment) among minoritized adolescents in the U.S. identified six classes of participants based on their growth trajectories during a four-year period (Huang & Stormshak, 2011). These classes were characterized by increases, stability, or decreases in ethnic-racial identity, and adolescents with increasing-high levels of ethnic-racial identity reported the best outcomes. More recently, a 2-year longitudinal study of Latinx and Black American adolescents in the U.S. found that both exploration and resolution significantly increased over time (Bañales et al., 2020). However, only resolution showed significant variability in the starting point and rate of change in these trajectories, and adolescents who demonstrated greater increases in resolution across the two years were more likely to have positive outcomes.

Albeit informative, studies based on variable-centered approaches may not fully capture the complexity of adolescents’ everyday life experience of ethnic-racial identity processes and how they develop over time, failing to account for fluctuations in identity at the individual level (Spiegler et al., 2019). In this perspective, a person-centered approach may be more appropriate to identify subgroups of individuals who share similar characteristics in terms of how their exploration and resolution evolve, as well as to better understand the relations between such subgroups and other predictors or outcome variables (van der Gaag, 2023). The current study used latent profile analysis to observe patterns of developmental trajectories of cultural identity process components and obtain a more nuanced description of their unique associations with psychosocial outcomes. Specifically, we focused on indices of positive development related to individual assets (global identity cohesion, self-esteem), interpersonal skills (other-group orientation, prosocial behavior), academic functioning (academic engagement), and mental health (depressive symptoms) given their previously reported links with ethnic-racial identity in adolescence (Umaña-Taylor & Rivas-Drake, 2021).

### Immigrant Background, Family Ethnic Socialization, and Adolescents’ Ethnic-Racial Identity Development

Ethnic-racial identity formation is shaped by individual and contextual factors (Phinney, 1993). Among these, membership in a minoritized group is particularly relevant in contexts characterized by a socially constructed ethnoracial hierarchy that determines differential access to resources and life opportunities (Umaña-Taylor, 2016). As posited by self-categorization theory, the salience of a certain group membership increases when the representation of one’s group in a certain context is low and there is unequal distribution of resources and power according to group membership (Turner et al., 1987). Prior research has shown that youth with minoritized/immigrant backgrounds tend to engage with their ethnic-racial identity earlier and more intensively than their majority/non-immigrant peers due to the heightened relevance of this component of the self (Erentaitė et al., 2018). This pattern is attributable to the complexity of identity formation for minoritized adolescents as they face issues of ethnoracial discrimination and the challenge of navigating multiple cultural value orientations (Schachner et al., 2018). Moreover, white normativity (Harlap & Riese, 2022) may lead individuals from the majority group to consider themselves as “normal” in comparison with racially minoritized individuals, resulting in less elaboration of, and reflection on, their heritage culture (Moffit & Juang, 2019). In support of this view, recent longitudinal work on ethnic-racial identity has found that adolescents with an immigrant background in Germany were more likely to be represented in high stable exploration and high-increase resolution profiles than their peers of non-immigrant descent (Hölscher et al., 2024).

Family ethnic socialization, that is, the implicit and explicit messages communicated within the family context on the importance of race and ethnicity and the implications of being of an ethnoracial minoritized group, also influences adolescents’ ethnic-racial identity formation (Hughes et al., 2006). Specifically, parents’ ethnic socialization efforts aimed at teaching youth about their cultural traditions, heritage, and pride contribute to the development of an increased sense of self-confidence and a more cohesive identity (Umaña‐Taylor and Hill (2020)), especially among ethnic-racial minoritized youth (Huguley et al., 2019). Several studies reported a positive association between family ethnic socialization and level of youth’s engagement in ethnic-racial identity processes (Gartner et al., 2014; Umaña-Taylor et al., 2013). A longitudinal study among Latinx adolescents in the U.S. examined classes of trajectories of ethnic-racial identity exploration and resolution across four years (Douglass & Umaña-Taylor, 2015), showing that youth characterized by high and increasing exploration and resolution reported the highest levels of family ethnic socialization. Another longitudinal study of Latinx youth in the U.S. across three years found that, whereas more family ethnic socialization experiences at baseline predicted stability of ethnic-racial identity exploration over time, they predicted less resolution development as participants progressed through adolescence (Constante et al., 2020).

### The *Identity Project* As a Way to Promote Ethnic-Racial Identity Development

Given the benefits of ethnic-racial identity exploration and resolution for the achievement of a coherent sense of self and psychological well-being, researchers developed the *Identity Project* intervention (Umaña-Taylor & Douglass, 2017). This school-based curriculum was designed to provide youth of any cultural background in the U.S. with tools and strategies to support them as they explore and seek to understand their constantly evolving identity in relation to race and ethnicity. Across 8 weekly sessions, trained facilitators engage participants in activities and reflections concerning personal and social identities, stereotypes and discrimination, meaningful cultural symbols, and family heritage. A randomized controlled trial demonstrated the efficacy of the program in enhancing students’ exploration from pre- to post-test, as well as a ripple effect of exploration on resolution at follow-up (Umaña‐Taylor et al. (2018a). A subsequent study reported that increased resolution predicted higher levels of global identity cohesion, self-esteem, and academic engagement and fewer depressive symptoms one year after pretest (Umaña-Taylor et al., 2018b). It was found that adolescents in the intervention group whose families had engaged in ethnic socialization practices to a greater extent prior to the intervention reported higher levels of exploration (Sladek et al., 2021). Moreover, different patterns of change emerged for resolution among majority youth compared to their peers with a minoritized background, such that they ended up at the same levels of resolution at the end of the study, but it took longer for White youth to achieve the same levels as youth of color (Sladek et al., 2021).

Based on these results, and given the increasing relevance of cultural identity-related topics in countries outside the U.S., the program has recently been adapted to and piloted in various multicultural societies in Europe (see Ceccon et al., 2024; Juang et al., 2020, 2022). Cultural adaptation was an essential process in light of the different historical backgrounds and sociocultural milieu that characterize many European countries as compared to the U.S. In the latter context, issues of ethnicity and race are particularly salient due to a socially constructed racial hierarchy that determines a privileged access to resources and life opportunities for the majority group (i.e., White), and marginalizes and systemically discriminates against other ethnoracial minoritized groups (i.e., Black, Latinx, Asian Americans, Native Americans; Umaña-Taylor, 2016). In Europe, a taboo still exists around the concept of “race” originating from World War II, during which the idea of alleged biological differences between groups led to legalized and nationwide discrimination, persecutions, and atrocities that are still vivid in people’s collective memory (Moffitt et al., 2020). Direct reference to this concept represents a sensitive topic, and the use of the word “race” is strongly discouraged in the local vocabulary (Juang et al., 2021). Furthermore, equating ethnic-racial identity and national identity, which is characteristic of European countries (Brubaker, 2009), contributed to a heightened stigmatization creating a dichotomous division between “natives” and “immigrants” in the public and scientific discourse (El-Tayeb, 2014). This has led several European scholars working with the *Identity Project* to use terms such as “cultural identity” or “heritage identity” when referring to ethnic-racial identity (Schotte et al., 2018), encompassing in this notion both the ethnic heritage and the racialization of a particular group in a given socio-historical context (Umaña‐Taylor et al. (2014)). It should be noted that, despite the different terminology, phenotypic traits and ethnic origin are still used as social markers (Jugert et al., 2022). Similarly, racialization as a societal system of power remains salient, increasing the relevance of ethnic-racial/cultural identity also for European youth (Erentaitė et al., 2018).

Implementations of the *Identity Project* in different European countries resulted in varying outcomes; for instance, evidence from Germany, Italy, and Sweden supported the efficacy of the program in enhancing adolescents’ exploration (Abdullahi et al., 2024; Ceccon et al., 2023; Juang et al., 2020), whereas the ripple effect of exploration on resolution was not supported in all countries (e.g., Ceccon et al., 2023; Schachner et al., 2024). As regards Italy, the intervention was well-received by schools due to the increasingly multiethnic composition of classrooms and teachers’ commonly experienced difficulty to address issues related to ethnicity, discrimination, and identity (Juang et al., 2022). Italy is a recent receiving society that shifted from being a country of emigration into one of immigration since the 1990s. It currently counts roughly 5 million legally residing individuals with non-Italian citizenship who make up 9% of the total population (ISTAT, 2023). Although over 200 different nationalities are represented, the largest immigrant communities originate from Romania, Albania, Morocco, China, and Ukraine. Within this context, the proportion of second-generation youth (i.e., individuals born in the host country from parents born abroad) amounts to 75% of all minors with an immigrant background (ISTAT, 2023). The heavily assimilationist attitude that characterizes the current socio-political climate in the country likely influences cultural identity formation processes in both minoritized and majority adolescents (Karataş et al., 2023). Hence, investigating these processes in a context where structured learning opportunities concerning ethnicity and culture occur less frequently in youth’s microsystems compared to countries with a longer history of immigration is essential to shed light on how exploration and resolution unfold, whether they are linked to immigrant background and socialization practices in the family, and if they relate to adjustment. The present study analyzed these processes in a sample of students who had participated in the intervention in school year 2022–2023 and for whom follow-up data were available one year after baseline. Due to the waitlist control design of the study, no data were available for the control group at the 1-year follow-up assessment because, by that time, all students had participated in the program.

## Current Study

Given the scarcity of person-centered approaches and the limited longitudinal research focusing on adolescents’ cultural identity in Europe, the present study examined trajectories of cultural identity processes and their associations with psychosocial outcomes among youth attending ethnically diverse schools in Italy. In doing so, the first aim was to examine unique and separate configurations of exploration and resolution trajectories in a sample of students who had participated in the *Identity Project*. Latent profile analysis was used to identify subgroups of adolescents based on their exploration and resolution levels assessed at four time points over a year. Considering prior theoretical and empirical work, the expectation was to find subgroups of adolescents showing increase, decrease, and stability in exploration and resolution processes. The second aim was to examine whether the emerging profiles would differ in terms of adolescents’ immigrant background and family ethnic socialization. Students with an immigrant background and those with higher levels of family ethnic socialization were expected to be more likely represented in profiles characterized by high-stable and/or increasing levels of exploration and resolution. The third aim was to study the associations between the emerging profiles and psychosocial functioning in the areas of global identity cohesion, self-esteem, academic engagement, depressive symptoms, other group orientation, and prosocial behavior. It was hypothesized that profiles with high-stable or increasing levels of resolution would exhibit the best psychosocial adjustment and, conversely, that profiles characterized by low-stable or decreasing levels of this variable would report the lowest levels of adjustment. As regards exploration, no hypothesis was made in light of theory suggesting that an increase in resolution (but not exploration) is linked to adolescents’ long-term psychological well-being within the *Identity Project*.

## Methods

### Participants and Procedure

After obtaining approval from the Ethics Committee of the School of Psychology at the University of Padova, the research team reached out to the principals and teachers with whom collaborations were already established during a large-scale implementation of the *Identity Project* conducted in the previous school year. The implementation was designed as a randomized, waitlist-control trial comprising three assessment points (pre-test, post-test, follow-up) with 747 students attending 45 classrooms (for a detailed description, see Ceccon et al., 2023). For the purpose of this study, the same schools were recontacted to conduct an additional, 1-year follow-up assessment. Out of the six public upper secondary schools originally involved in the project, one did not respond, one declined due to a lack of time and resources supporting the logistics of data collection, and one had a specific academic organization in which students chose their preferred specialization at the beginning of the school year, resulting in a completely new classroom composition that rendered it difficult to trace back participants from the intervention group of the previous year. The remaining three schools were available to take part in the study. Schools who did (*n* = 3) and did not participate (*n* = 3) in the study did not differ in the proportion of youth with an immigrant background (*χ*^*2*^ = 0.11, *df* = 1, *p* = 0.751, Cramer’s *V* = 0.01), students’ socioeconomic status (*t* = −0.74, *df* = 742, *p* = 0.461, Cohen’s *d* = −0.05), age (*t* = 1.05, *df* = 742, *p* = 0.293, Cohen’s *d* = 0.08), or gender (*χ*^*2*^ = 5.43, df = 1, *p* = 0.07, Cramer’s *V* = 0.08). Next, participants were recruited among those students who had been randomly assigned to the intervention group in these three schools (*n* = 268). Written informed consent forms to be signed by parents and adolescents were distributed to 219 students in total; 49 eligible students had either failed the year or changed class/school, and therefore could not be involved. For ethical reasons, one student with an immigrant background whose Italian proficiency was insufficient to complete the survey was nonetheless invited to participate in the assessment with the help of a facilitator/support teacher, but responses were not considered for subsequent analyses. Two-hundred fourteen out of the 219 students who had received consent forms agreed to participate in the follow-up assessment, with a participation rate of 98%. Questionnaires measuring the variables of interest (see Measures section) were completed during school hours by a total of 206 students (8 were absent from school on the day of survey administration) in the presence of the teacher and two facilitators. The participants filled in the survey via an online platform (i.e., Qualtrics) and could choose among Italian, Chinese, and English language (these specific versions were made available based on participants’ previous requests).

For analytic purposes, the final sample was composed of students in the intervention group whose data were available for all four assessments: Time 0 (T0; baseline), 1 week prior to the intervention; T1 (9-week post-test); T2 (13-week post-test) and T3 (54-week post-test), resulting in *N* = 173 adolescents (*M*_age_ = 14.99, *SD* = 0.62, range = 14–17 years old; 58.4% female, 39.3% male, 2.3% non-binary). Data from this sample are available in the Open Science Framework at the following link: https://osf.io/maf4q/?view_only=d71fd907bb3c4dbe804b926b7cfa1c08.

To test whether participants in the intervention group who attended (*n* = 173) and who did not attend all the survey administrations (*n* = 332) differed at baseline, a logistic regression model was performed including sociodemographic characteristics (i.e., gender, immigrant background, and socioeconomic status) and the main variables of interest (i.e., cultural identity exploration and resolution) as predictors, and having participated in the four assessments as the dependent variable. The analysis of deviance showed that no predictors had a significant effect except for gender (*χ*^*2*^ = 8.26, *df* = 1, *p* = 0.004), with the group of students who participated in all assessments including more girls (60%) than the group of students who missed at least one assessment (46%)^1^.

With respect to sociodemographics, 26% of the participants had an immigrant background (i.e., were born abroad or in Italy from at least one parent born abroad); of these, 75% were born in Italy (i.e., second-generation). First-generation youth had lived in Italy for 6 years on average (*SD* = 2.87, range = 2–10 years). Overall, adolescents reported 18 different countries of origin (also including their parents’ birth countries), the main ones being Moldova, Romania, Albania, Morocco, and Tunisia. One percent of parents had completed primary school, 18% had completed lower secondary school, 53% had completed upper secondary school, 27% attended university, and 1% were missing or preferred not to answer/did not know. As regards socioeconomic status, the mean score on the Family Affluence Scale (see Measures section) was 6.43 (*SD* = 0.62, range = 2–9). The demographic composition of the participating schools and the descriptives of study variables at baseline are reported in Table A.1, Appendix A1, Online Supplementary Material.

### Measures

#### Sociodemographics

Students reported on their age, gender, country of birth (both their own and their parents’), length of residence in Italy (for students born abroad), and parental educational level. Immigrant background was coded as 0 (born in Italy from Italian-born parents) or 1 (born in Italy or abroad from at least one parent born abroad) following previous research (see Schachner et al., 2016).

#### Socioeconomic status

This variable was assessed through the Family Affluence Scale (Currie et al., 2008), which comprises 4 items measuring family wealth (e.g., “Does your family have a car?”). The total score is calculated by summing scores given to each item and ranges from 0 (lowest affluence) to 9 (highest affluence): scores between 0 and 2 reflect low affluence, 3 to 5 medium affluence, and 6 to 9 high affluence. This instrument had been previously used among Italian adolescents, showing support for construct validity (Boyce et al., 2006).

#### Cultural identity exploration and resolution

An adapted version of the exploration (7 items, e.g., “I have participated in activities that have exposed me to my culture of origin”) and resolution (4 items, e.g., “I understand how I feel about my culture of origin”) subscales from the Ethnic Identity Scale (Umaña-Taylor et al., 2004) were used to measure the two main variables of interest. Response options range from 1 (does not describe me at all) to 4 (describes me very well); total scores are obtained by averaging item responses of each subscale, with higher scores representing higher levels of each dimension. Although the original version of the scale referred to “ethnic” identity, in the Italian adaptation, students were asked to write down their heritage culture (i.e., cultural background prevalent in their family) before rating the items, and answered the subsequent questions referring to that specific cultural group. This specification was included because during the *Identity Project*, adolescents with bi/multi-cultural backgrounds are encouraged to especially explore their heritage culture (i.e., “foreign” culture as opposed to the Italian one), because it is likely that this part of their identity/background is the most stigmatized one (by society in general, teachers, peers, etc). However, to convey the idea that we all can identify with more than one cultural group and that they would not be asked to choose just one among their cultural affiliations during the activities, at the end of the questionnaire, students could then also list all other cultural groups with which they self-identified. The questionnaire has been previously used among multiethnic youth in the U.S. and Italy, demonstrating good validity and invariance across adolescents with and without an immigrant background (Ceccon et al., 2023; Sladek et al., 2020). In the present study, Cronbach’s alphas and McDonald’s omegas were *α* = 0.77, 95% CI [0.71–0.82] and *ω* = 0.79, 95% CI [0.73–0.83] for the exploration subscale, and *α* = 0.82, 95% CI [0.76–0.86] and *ω* = 0.82, 95% CI [0.78–0.87] for the resolution subscale.

#### Family ethnic socialization

This variable was assessed through the Familial Ethnic Socialization Measure (Umaña-Taylor et al., 2004). The 12 items (e.g., “My family teaches me about our cultural background”) are scored on a 5-point Likert scale from 1 = not at all to 5 = very much; the total score is obtained by averaging all item responses, with higher scores indicating higher levels of family ethnic socialization. The scale has shown good reliability and validity across culturally diverse adolescent samples (Sladek et al., 2020). In this study, Cronbach’s alpha was *α* = 0.88, 95% CI [0.84–0.90], and McDonald’s omega was *ω* = 0.88, 95% CI [0.85–0.91].

#### Global identity cohesion

This construct was assessed via the identity subscale of the Erikson Psychosocial Stage Inventory (Rosenthal et al., 1981), which comprises 12 items (e.g., “I have a clear idea of who I want to be”) rated on a response scale from 1 = strongly disagree to 5 = strongly agree. Items forming the subscale are averaged to obtain the total score, with higher scores indicating greater cohesion. This instrument has been widely used among diverse adolescent samples from various countries, including Italy (Dimitrova et al., 2018). In this study, Cronbach’s alpha was *α* = 0.85, 95% CI [0.81–0.88], and McDonald’s omega was *ω* = 0.85, 95% CI [0.82–0.88].

#### Self-esteem

The Rosenberg Self-Esteem Scale (Rosenberg, 1979) was used to measure participants’ self-esteem. This scale includes 10 items (e.g., “I feel that I have a number of good qualities”) rated on a 5-point Likert scale (1 = strongly disagree to 4 = strongly agree); total scores are obtained by summing scores for each item. The scale has been validated in Italy (Prezza et al., 1997), showing good internal consistency, external validity, and invariance across cultural groups (Li et al., 2015). In this study, Cronbach’s alpha was *α* = 0.91, 95% CI [0.89–0.92], and McDonald’s omega was *ω* = 0.92, 95% CI [0.90–0.93].

#### Depressive symptoms

The brief version of the Center for Epidemiological Studies Depression Scale (Radloff, 1977) was used to assess depressive symptoms. The scale comprises 10 items (e.g., “My sleep was restless”) rating the frequency of depressive symptoms from 0 (rarely or none of the time) to 3 (most of the time) in the past week. Scores assigned to each item are summed to obtain the total score. The factorial validity and psychometric properties of the brief version of this instrument have been previously demonstrated in studies involving adolescent samples (Bradley et al., 2010). In this study, Cronbach’s alpha was *α* = 0.86, 95% CI [0.82 - 0.89], and McDonald’s omega was *ω* = 0.86, 95% CI [0.83–0.89].

#### Academic engagement

Participants’ behavioral and emotional engagement in school activities was assessed via the Engagement vs. Disaffection with Learning: Student Report Scale (Skinner et al., 2008). The questionnaire includes 10 items (e.g., “When I am in class, I listen very carefully”) rated on a 5-point Likert scale from 0 (never) to 4 (all the time); item scores are averaged to obtain the total score, with higher scores indicating greater engagement. Studies reported a strong correlation between the student and teacher forms of the scale, supporting its construct validity (e.g., Skinner et al., 2008). In this study, Cronbach’s alpha was *α* = 0.92, 95% CI [0.89–0.93], and McDonald’s omega was *ω* = 0.92, 95% CI [0.90–0.94].

#### Other group orientation

Attitudes toward other cultural groups were measured by the 6-item Other-Group Orientation subscale (e.g., “I like meeting and getting to know people from cultural groups other than my own”) of the Multigroup Ethnic Identity Measure (Phinney, 1992). Response options range from 1 (strongly disagree) to 4 (strongly agree), with items being averaged to obtain the total score; higher scores indicate higher levels of the variable. Previous studies with ethnoracially diverse samples provided evidence for reliability and validity for this subscale (Sladek et al., 2020). In this study, Cronbach’s alpha was *α* = 0.74, 95% CI [0.66–0.79], and McDonald’s omega was *ω* = 0.75, 95% CI [0.69–0.82].

#### Prosocial behavior

Students completed the Friends subscale from the adapted version (Padilla‐Walker et al. (2018)) of the Kindness and Generosity Inventory of Strengths (Peterson & Seligman, 2004). This subscale includes 9 items (e.g., “I voluntarily help my friends”) rated on a 5-point Likert scale from 1 (not at all like me) to 5 (very much like me). Item scores are averaged to obtain the total score, with higher scores indicating greater prosociality toward friends. In this study, Cronbach’s alpha was *α* = 0.85, 95% CI [0.81–0.88], and McDonald’s omega was *ω* = 0.85, 95% CI [0.82–0.88], in line with previous research showing good internal validity of this instrument (Mesurado et al., 2019).

### Data Analysis

All statistical analyses were performed using *R* software (*R* Core Team, 2018). Descriptive statistics and bivariate correlations are shown in Table 1. To address the first aim, a model-based longitudinal latent profile analysis was conducted using the *mclust* package of the *R* software (Scrucca et al., 2016). For each dependent variable, i.e., cultural identity exploration and resolution, several clustering models were tested (for a detailed explanation of the clustering models, see Scrucca et al., 2016). The best fitting model was selected using the BIC index, which indicates the plausibilty of a model based on the observed data (Raftery, 1995). Specifically, when using the package *mclust*, the BIC index is calculated so that the best model is the one with the highest score (e.g., between two models with scores -95 and -100, the best would be the one associated to the -95 score; see also Giofrè et al., 2019). To account for potential school effects, Pearson’s chi-squared test was used to examine whether school was associated with profile membership. To address the second aim, Pearson’s chi-squared test was used to examine whether adolescents’ immigrant background was associated with profile membership, whereas a linear regression model was used to evaluate whether the emerging profiles differed in terms of family ethnic socialization. To address the third aim, separate multiple linear regression models were performed for each of the six psychosocial outcomes (i.e., global identity cohesion, self-esteem, academic engagement, depressive symptoms, other group orientation, and prosocial behavior), including profile membership as the predictor and each psychosocial outcome measured at T3 (1-year follow-up) as a dependent variable, controlling for the same variable at T0 (baseline), school, immigrant background, gender, and age. These latter sociodemographic variables were included as covariates following previous research in the area of ethnic-racial/cultural identity development (e.g., Umaña‐Taylor et al. (2018a).

**Table 1:** Correlations and Descriptive Statistics for Study Variables (*N* = 173)

	1.	2.	3.	4.	5.	6.	7.	8.	9.	10.	11.	12.	13.	14.	15.	16.	17.	18.	19.	20.	21.	22.	23.	24.	25.
1. T0 EXPL		0.58***	0.54***	0.44***	0.53***	0.35***	0.33***	0.32***	−0.01	0.06	0.09	0.08	0.05	−0.03	0.28***	0.23**	0.23**	0.19*	0.16*	0.23**	0.56***	0.18*	0.22**	0.01	−0.03
2. T1 EXPL			0.70***	0.57***	0.28***	0.38***	0.28***	0.30***	−0.05	0.06	0.05	0.07	0.13	0.11	0.09	0.20**	0.25***	0.26***	0.17*	0.12	0.52***	0.16*	0.21**	0.01	0.02
3. T2 EXPL				0.67***	0.36***	0.33***	0.45***	0.40***	0.02	0.08	0.08	0.15	0.06	0.06	0.16*	0.24**	0.23**	0.23**	0.13	0.21**	0.49***	0.32***	0.14	−0.01	−0.06
4. T3 EXPL					0.35***	0.40***	0.44***	0.56***	0.10	0.24**	0.19*	0.30***	−0.01	−0.08	0.08	0.26***	0.25***	0.39***	0.13	0.23**	0.49***	0.35***	0.09	0.03	−0.02
5. T0 RES						0.52***	0.56***	0.52***	0.16*	0.27**	0.20**	0.22**	−0.13	−0.20**	0.17*	0.19*	0.17*	0.14	0.05	0.09	0.39***	0.19*	0.03	0.12	−0.02
6. T1 RES							0.64***	0.55***	0.25***	0.32***	0.27***	0.32***	−0.13	−0.13	0.03	0.22**	0.08	0.13	0.12	0.21**	0.29***	0.15*	−0.03	0.01	−0.01
7. T2 RES								0.65***	0.16*	0.35***	0.20**	0.35***	−0.14	−0.20**	0.10	0.22**	0.07	0.10	0.07	0.19*	0.35***	0.17*	−0.01	0.06	0.01
8. T3 RES									0.20**	0.39***	0.19*	0.36***	−0.12	−0.19*	0.05	0.31***	0.18*	0.28***	0.11	0.30***	0.38***	0.29***	0.03	−0.02	0.01
9. T0 GIC										0.61***	0.72***	0.52***	−0.62***	−0.38***	0.07	0.31***	−0.03	−0.02	−0.13	−0.02	0.06	−0.04	−0.30***	−0.01	0.16*
10. T3 GIC											0.57***	0.79***	−0.47***	−0.58***	0.10	0.37***	0.02	0.15*	−0.06	0.01	0.06	0.05	−0.14	−0.01	0.08
11. T0 S−E												0.65***	−0.76***	−0.53***	0.01	0.24**	−0.04	0.05	−0.17*	−0.10	−02	−0.05	−0.36***	−0.03	0.14
12. T3 S−E													−0.52***	−0.59***	0.04	0.36***	0.02	0.13	−0.05	0.05	0.08	0.14	−0.19*	−0.02	−0.09
13. T0 DEPS														0.65***	0.07	−0.14	0.16*	0.07	0.29***	0.16*	0.06	0.11	0.46***	0.05	−0.13
14. T3 DEPS															−0.04	−0.27**	−0.01	−0.04	0.20**	0.10	0.01	0.07	0.28***	−0.08	0.01
15. T0 ENG																0.48***	0.09	0.04	0.21**	0.25***	0.11	0.02	0.31***	0.09	−0.05
16. T3 ENG																	0.19*	0.19*	0.19*	0.26***	0.07	0.17*	0.18*	−0.01	−0.05
17. T0 OGO																		0.50***	0.17*	0.25***	0.15*	0.23**	0.23**	0.02	−0.08
18. T3 OGO																			0.03	0.12	0.15	0.25***	0.23**	−0.09	−0.11
19. T0 PROSB																				0.53***	0.16*	−0.01	0.22**	−0.18*	0.13
20. T3 PROSB																					0.17*	0.09	0.20*	−0.08	0.02
21. FES																						0.26***	0.11	0.08	−0.03
22. IMMIGR																							0.18*	0.17*	−0.33***
23. Gender																								0.04	−0.07
24. Age																									−0.25**
25. SES																									
*M (SD)*	2.62 (0.56)	2.73 (0.51)	2.67 (0.55)	2.64 (0.59)	2.79 (0.62)	2.92 (0.60)	2.92 (0.59)	2.95 (0.61)	3.25 (0.65)	3.20 (0.50)	25.73 (6.88)	26.67 (6.10)	14.15 (6.38)	12.50 (5.63)	2.28 (0.61)	1.75 (0.51)	3.34 (0.46)	3.24 (0.55)	3.88 (0.59)	3.78 (0.72)	2.97 (0.69)	0.26 (0.44)	1.60 (0.49)	14.99 (0.62)	6.43 (1.72)

Immigrant background was coded as 0 = without immigrant background (i.e., born in Italy from Italian-born parents) and 1 = with immigrant background (i.e., born in Italy or abroad from at least one parent born abroad). Gender was coded as 1 = males and 2 = females. T0 = pretest, T1 = 9-week posttest, T2 = 13-week follow-up, T3 = 54-week follow-up

*EXPL* Cultural identity exploration, *RES* cultural identity resolution, *GIC* Global identity cohesion, *S-E* Self-esteem, *DEPS* Depressive symptoms, *ENG* Academic engagement, *OGO* Other group orientation, *PROSB* Prosocial behavior, *FES* Family ethnic socialization, *IMMIGR* Immigrant background. *SES* Socioeconomic status

*p < 0.05, **p < 0.01, ***p < 0.001

In all above-mentioned models, when a significant effect of profile membership emerged, pairwise comparisons between profiles were conducted using the Tukey method in order to control for Type I error via the *emmeans* package (Lenth, 2023; *R* Core Team, 2018). Partial eta squared was used as a measure of effect size and interpreted according to widely accepted guidelines (Cohen, 1969; Funder & Ozer, 2019).

## Results

### Preliminary Analyses

In preliminary analyses, missing data in the analytic sample (*n* = 173) across the four time points were screened; overall, missing data ranged from 0.00% to 0.53% (mean = 0.27%) across study variables. For each participant, missing values were imputed based upon each subject’s mean score on each subscale of a given measure. This type of single imputation was selected given the well-established and validated questionnaires used in this study, as well as the extremely low proportion of missing values in our data (< 1%; see Garson, 2015).

### Longitudinal Profiles of Cultural Identity Exploration and Resolution

**Fig. 1:**
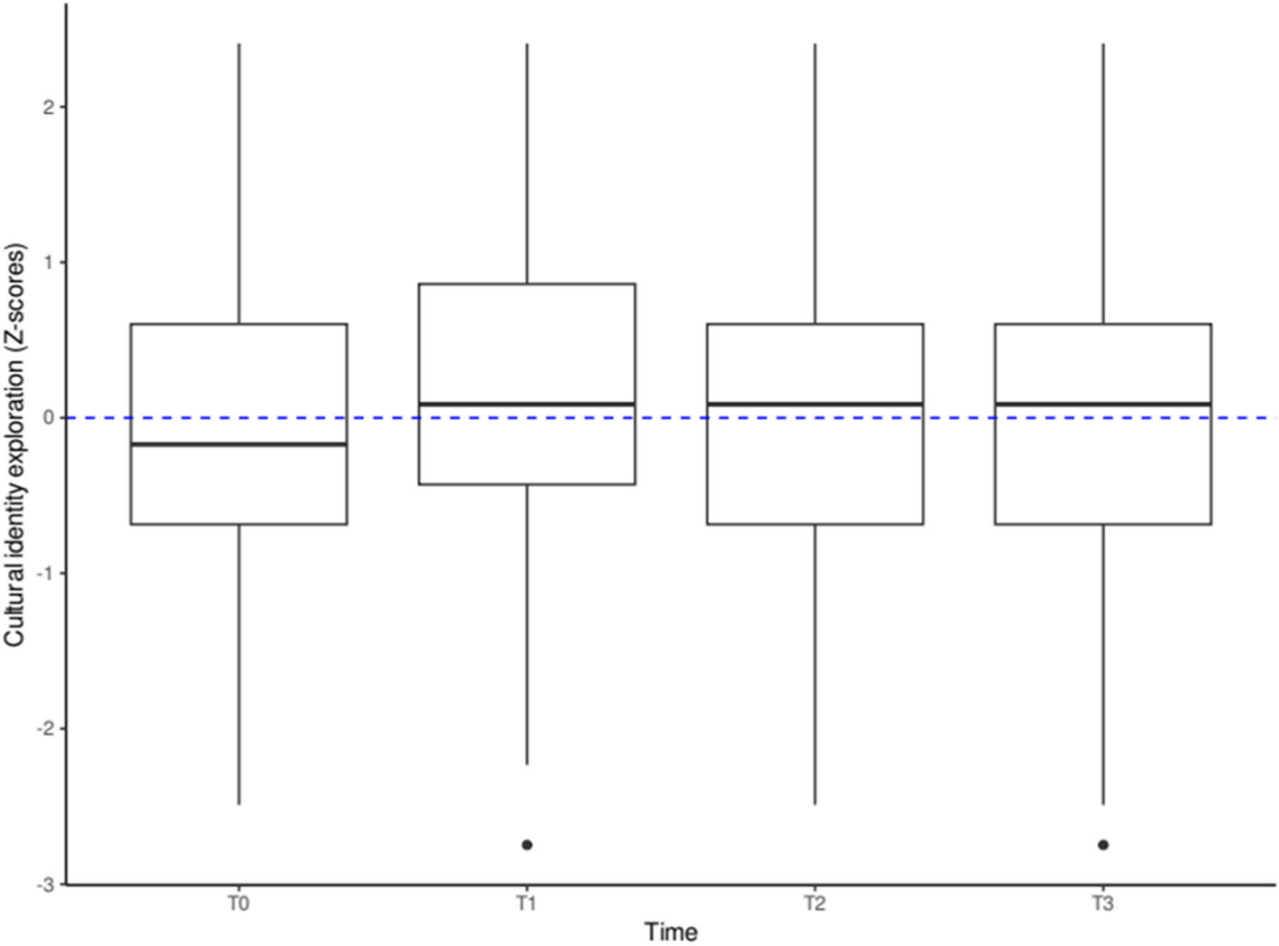
Profile of cultural identity exploration

For cultural identity exploration, the most plausible model (BIC = −903.1184) identified one single profile. A visual representation of the trend over time of cultural identity exploration is depicted in Fig. 1: students in this profile showed an increase in exploration from T0 to T1, a decrease at T2, and returned to initial levels of exploration at T3.

**Fig. 2:**
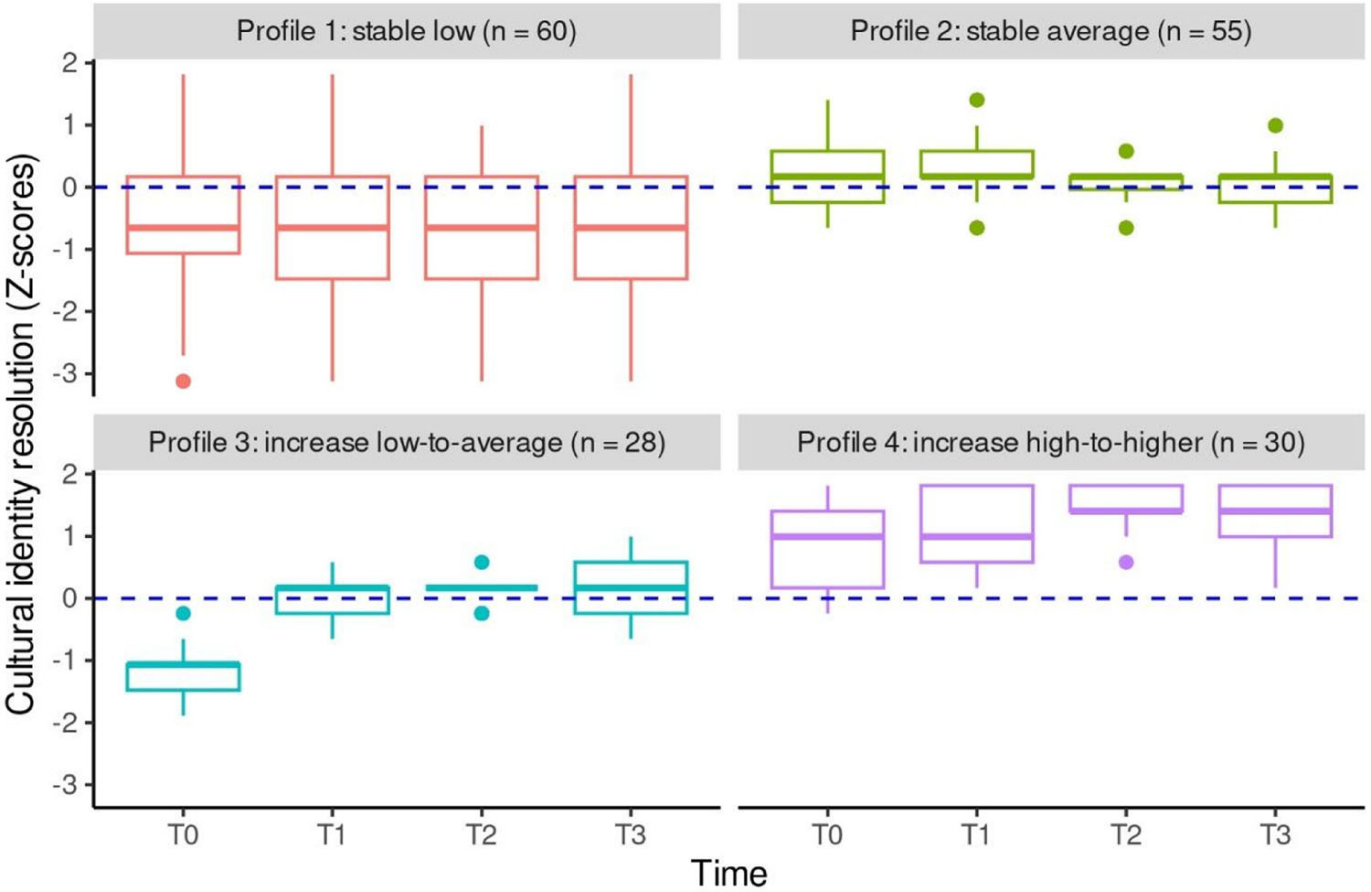
Profiles of cultural identity resolution

For cultural identity resolution, the most plausible model (BIC = −1007.337) identified four different profiles. A visual representation of the trend over time of each of these profiles can be found in Fig. 2. Participants in profile 1 (“stable low”, *n* = 60, 35%) remained stable at low levels of resolution; participants in profile 2 (“stable average”, *n* = 55, 32%) also remained stable, but at medium levels of resolution; participants in profile 3 (“increase low-to-average”, *n* = 28, 16%) increased over time in their levels of resolution, starting from a low level and reaching an average level; participants in profile 4 (“increase high-to-higher”, *n* = 30, 17%) started from a high level of resolution and showed a further increase. Given the presence of only one profile for exploration, subsequent analyses exclusively focused on the four resolution profiles. No significant association was detected between school and profile membership (*χ*^2^ = 5.55, *df* = 6, *p* = 0.475, Cramer’s *V* = 0.13); hence, the schools did not differ in the distribution of the resolution profiles.

### Associations of Immigrant Background and Family Ethnic Socialization With Resolution Profiles

The chi-squared test revealed a significant association between immigrant background and membership in the resolution profile, *χ*^*2*^ = 18.18, *df* = 3, *p* < 0.001, Cramer’s *V* = 0.32. Students with an immigrant background were overrepresented (57%) in the “increase high-to-higher” profile, and slightly underrepresented (17%) in the “stable low” profile; the ratio between students with and without an immigrant background in the “stable average” (22% vs 78%) and “increase low-to-average” (21% vs 79%) profiles mirrored the one in the general sample (26% vs 74%).

The linear regression model showed a significant association between profile membership and family ethnic socialization, *F*(3,169) = 12.475, *p* < 0.001, *η*_*p*_^2^ = 0.18. Detailed results of the model are presented in Table A.1, Appendix A1, Online Supplementary Material. Pairwise comparison between profiles via the Tukey method showed that adolescents in the “increase high-to-higher” profile reported significantly higher levels of family ethnic socialization than their peers in the “stable-low” (*t*(169) = −5.348, *p* < 0.001) and in the “increase-low-to-average” profile (*t*(169) = −2.705, *p* = 0.037). Students in the “stable-average” profile reported significantly higher levels of family ethnic socialization than their peers in the “stable-low” profile (*t*(169) = −4.820, *p* < 0.001). As shown in Figure A.1, Appendix A2, Online Supplementary Material, participants in the “increase high-to-higher” profile exhibited the highest level of family ethnic socialization, whereas the ones in the “stable low” profile reported the lowest level of this variable.

### Associations Between Resolution Profiles and Psychosocial Outcomes

All detailed results of each of the six multiple regression models are presented in Tables A.3–A.8, Appendix A3, Online Supplementary Material. Multiple linear regression models^2^ showed a significant and medium effect of profile membership on global identity cohesion (*F*(3,159) = 7.20, *p* < 0.001, *η*_*p*_^2^ = 0.12), self-esteem (*F*(3,159) = 6.05, *p* < 0.001, *η*_*p*_^2^ = 0.10), and prosocial behavior (*F*(3,159) = 5.45, *p* = 0.001, *η*_*p*_^2^ = 0.09), a significant and small effect on depressive symptoms (*F*(3,159) = 3.00, *p* = 0.032, *η*_*p*_^2^ = 0.05), and no significant effect on academic engagement (*F*(3,159) = 1.68, *p* = 0.174, *η*_*p*_^2^ = 0.03) and other group orientation (*F*(3,159) = 0.65, *p* = 0.583, *η*_*p*_^2^ = 0.01).

Follow-up pairwise comparisons via the Tukey method indicated that students in the “increase high-to-higher” profile scored significantly higher on global identity cohesion than their counterparts in the “stable-low” (*t*(168) = −4.981, *p* < 0.001) and “stable-average” profiles (*t*(168) = −4.169, *p* < 0.001); these students also reported greater self-esteem than their peers in the “stable-low” (*t*(168) = −4.706, *p* < 0.001) and “stable-average” profiles (*t*(168) = −4.001, *p* < 0.001). Adolescents in the “increase low-to-average” profile reported significantly higher self-esteem compared to their peers in the “stable-low” profile (*t*(168) = −2.863, *p* = 0.024). Youth in the “increase-high-to-higher” profile scored significantly lower on depressive symptoms than their counterparts in the “stable low” profile (*t*(168) = 2.720, *p* = 0.036), reported more academic engagement than youth in the latter profile (*t*(168) = −3.210, *p* = 0.009), and engaged in more prosocial behavior than their peers in the “stable-low” (*t*(168) = −3.120, *p* = 0.011) and “stable-average” profiles (*t*(168) = −3.332, *p* = 0.006).

Overall, as shown in Fig. 2, Appendix A3, Online Supplementary Material, participants in the “increase high-to-higher” profile reported the best psychosocial outcomes for all variables one year after participating in the intervention, followed by participants in the “increase low-to-average” profile (except for depressive symptoms, with participants in this profile reporting approximately the same levels as their peers in the “stable-average” profile). Conversely, participants in the “stable-low” profile exhibited the worst psychosocial outcomes for all indicators except for prosocial behavior (the lowest levels of this variable were observed in the “stable-average” profile).

## Discussion

Although extant evidence indicates that reaching a definition of one’s identity in relation to one’s cultural background(s) is a crucial developmental skill in diverse societies, longitudinal research adopting a person-centered approach to investigate cultural identity in the European context is still limited. Addressing this gap in the literature, this study aimed to provide novel evidence concerning the unfolding of cultural identity processes and their potential associations with psychosocial adjustment in the long run among adolescents living in Italy. In doing so, the study set out to identify profiles of trajectories of cultural identity exploration and resolution among ethnically diverse students who had participated in the *Identity Project* (Umaña-Taylor & Douglass, 2017), a school-based intervention especially designed to boost these processes. Overall, the findings revealed that there was substantial heterogeneity in how adolescents evolved in terms of resolution, but not exploration. Students with immigrant background and high levels of family ethnic socialization were more likely to belong to the group characterized by an increase in resolution over time starting from high levels of this variable. Furthermore, adolescents in the latter group were those who showed the highest levels of psychosocial adjustment in the long run, followed by their peers who increased from low to average levels of resolution.

In relation to the first aim, longitudinal latent profile analysis identified one exploration profile and four resolution profiles. Exploration increased from T0 to T1, decreased at T2, and returned to initial levels at T3. Hence, no subgroups of students emerged in relation to how this process evolved over time. This finding was somewhat unexpected in light of prior research identifying different trajectories of exploration in adolescence. However, it should be noted that the results from the current study are not directly comparable with previous studies using different statistical approaches (e.g., parallel process group-based trajectory modeling; see Douglass & Umaña-Taylor, 2015) or cross-sectional data (e.g., Meca et al., 2023). Moreover, the current sample was composed of youth who had participated in a school-based intervention. In this perspective, the fact that all participants exhibited the same developmental trajectory in terms of exploration might indicate that the intervention was equally salient and efficacious in stimulating an in-depth search and observation of their heritage culture(s) for all youth, regardless of their background. The identification of a single profile supports the “universality” of the *Identity Project*, as intended and specifically designed by its developers (see Umaña-Taylor & Douglass, 2017). The increase in exploration from pre- to posttest further confirms the results of efficacy studies of the *Identity Project* conducted in Italy and the U.S. (Ceccon et al., 2023; Umaña‐Taylor et al. (2018a).

As regards resolution, latent profile analysis revealed a more nuanced pattern, with students in the first and second profiles showing stability (at a low and average level, respectively), and those in the third and fourth profiles substantially increasing over time (one from a low to average level, and one from a high to higher level). The identification of four profiles for resolution characterized by both stability and change is consistent with recent findings from the German (Hölscher et al., 2024) and Swedish (Abdullahi et al., 2024) implementations of the *Identity Project*, and resembles previous research on profiles of trajectories of ethnic-racial identity content among Black college students in the U.S. (Chavous et al., 2018). The heterogeneity of resolution profiles emerging from the current study supports the notion that achieving a sense of clarity of one’s cultural identity and the meaning assigned to it is an introspective and private process that might be more subject to individual differences. Notably, the absence of profiles with a decreasing trend among students who had participated in the *Identity Project* suggests that the intervention encouraged, or at least sustained, an ongoing reflection and awareness around cultural identity. It is also worth mentioning that, for the two increasing profiles, only participants in the “increase high-to-higher” profile exhibited the highest levels of resolution at T2 (first follow-up), while the ones in the “increase low-to-average” profiles showed an increase already at T1 (post-test). This might explain why the ripple effect on resolution was not found in the Italian study (Ceccon et al., 2023). On the other hand, employing a person-centered approach enabled us to discover that this effect indeed occurred for a subgroup of participants, following the original theoretical model and the U.S. implementation of the *Identity Project* (Umaña‐Taylor et al. (2018a).

In relation to the second aim, associations of adolescents’ immigrant background and levels of family ethnic socialization with profile membership were examined based on previous research showing how both variables often predict a stronger cultural identity (Umaña‐Taylor and Hill (2020). In the first three resolution profiles (“stable low”, “stable average”, “increase low-to-average”), the ratio between students with and without immigrant background roughly mirrored the one in the general sample, with the relative highest percentage of students without immigrant background being in the “stable low” profile. Conversely, youth with an immigrant background were overrepresented in the fourth profile (i.e., “increase high-to-higher”). In line with previous work (Erentaitė et al., 2018), this pattern supports the idea that minoritized youth were more engaged with cultural identity formation processes from the beginning of the intervention, but they nonetheless showed an increase over time which was then linked to better psychosocial outcomes. As regards their non-immigrant peers in the same longitudinal profile, the high (and increasingly high) levels of resolution may partly be explained by the current Italian sociopolitical climate emphasizing nationalism and protection of Italian heritage identity, although it should be noted that resolution does not include the dimension of positive affect towards one’s own cultural identity (Umaña-Taylor et al., 2004). An alternative explanation is that these students had elaborated a personal interpretation of what being Italian means to them not necessarily aligned with a nationalist model of “Italianness” typical of previous generations (Dixon et al., 2018). In support of this view, during the sessions and focus groups conducted after the end of the program (see Ceccon et al., 2023, 2024), many Italian-origin students expressed appreciation for having the possibility to critically reflect on the various elements of their culture (e.g., arts, history, food) in a group setting in which the heritage of other cultures was equally received in a welcoming way. This suggests a more nuanced representation of the multiple dimensions constituting their cultural identity (e.g., religion, nation, region, local dialect) as well as an openness toward individuals with different cultural backgrounds.

Regarding family ethnic socialization, an association with profile membership emerged, with youth in the “increase high-to-higher” profile reporting the highest level of family ethnic socialization at baseline. Hence, it is possible that the increase in resolution over time was driven by participation in the *Identity Project* program. As regards the two stability profiles, students in the “stable low” profile reported less family ethnic socialization than their peers in the “stable average” profile. Given that family ethnic socialization has been shown to be promotive for ethnic-racial identity (Umaña‐Taylor and Hill (2020), the different baseline levels of resolution (low vs average) characterizing the two profiles might be due to the higher levels of family ethnic socialization exhibited by participants in the “stable average” (vs “stable low”) profile. Overall, the overrepresentation of adolescents with an immigrant background and who had experienced the highest levels of ethnic socialization in the family prior to participating in the program in the profile marked by high initial levels of resolution and a further increase (i.e., “high-to-higher” profile) aligned with our expectations, supporting previous evidence that these two variables are linked to adolescents’ cultural identity. This type of social identity is especially meaningful and salient for youth from ethnoracially minoritized backgrounds (Umaña-Taylor & Rivas-Drake, 2021), and is modeled by the messages individuals receive within the home environment in relation to their cultural traditions and heritage (Hughes et al., 2006). This finding further implies that the *Identity Project* intervention, in combination with high levels of family ethnic socialization, still played an important role in supporting youth to gain clarity with respect to their cultural background(s), as shown by the increase in resolution over time.

The third aim concerned the associations among resolution profiles and a host of psychosocial outcomes (i.e., global identity cohesion, self-esteem, academic engagement, depressive symptoms, other group orientation, and prosocial behavior) assessed among participants one year after they had started the *Identity Project* intervention. Profile membership emerged as a significant predictor of all outcomes, except for academic engagement and other group orientation. In particular, youth in the “increase high-to-higher” profile exhibited the best psychosocial adjustment at the one year follow-up, followed by those who were in the “increase low-to-average” profile. On the contrary, adolescents in the profile showing a low stable trajectory of resolution were the ones exhibiting the worst outcomes in terms of adjustment. These findings are in line with previous studies demonstrating the promotive role of ethnic-racial identity in academic and psychological adjustment (see Umaña-Taylor & Rivas-Drake, 2021). Specifically, the results mirror those emerging from the longitudinal study conducted in the U.S. among youth participating in the *Identity Project*, where increases in ethnic-racial identity processes were associated with better psychological and academic adjustment (i.e., greater global identity cohesion and self-esteem, lower depressive symptoms, and higher grades) at the follow-up assessment one year later (Umaña-Taylor et al., 2018b). In the current study, no long-term effects on students’ involvement toward classroom activities and attitudes toward people from other cultural groups emerged (see also Sandberg et al., 2024). However, also in the original efficacy study, ethnic-racial identity was positively associated with these two variables (i.e., other group orientation and academic engagement) only when global identity cohesion was examined as a mediator of program effects (Umaña-Taylor et al., 2018b). Future research might investigate possible indirect effects of ethnic-racial identity on intergroup attitudes and school involvement through its impact on adolescents’ overall sense of self-concept and synthesis/cohesion of their global identity.

Several limitations should be considered when interpreting the results. First, the relatively small sample size may have influenced the number of extracted profiles, especially in the case of cultural identity exploration, as well as the comparisons across subgroups of adolescents. Relatedly, the modest size of the profiles and the heterogeneity of nationalities in the current sample did not allow to further examine whether the profiles were differently characterized based on students’ national origin or generational status (i.e., first vs second generation). Future longitudinal studies with larger samples of adolescents are needed to provide a more nuanced picture of individual differences in exploration and resolution processes over time, accounting for other relevant individual and contextual factors that may influence such processes. Second, the waitlist control design prevented us from comparing students in the intervention and control group, because the latter had received the *Identity Project* after the first follow-up (T2). Nevertheless, the current study’s focus on adolescents in the intervention group provides important insights into how specific profiles are linked to long-term psychosocial outcomes, and the identification of varied resolution profiles can inform on the potential need and best timing for the implementation of booster sessions to keep youth at high levels of this process. Third, this study reflected participants’ identity only in terms of heritage culture identity. Following extant research considering both national and ethnic-racial identity (Meca et al., 2023), future studies should explore whether, for bicultural or multicultural adolescents, their national identity follows a different developmental trajectory and is differently associated with psychological and academic outcomes. This might particularly apply to youth, like those in this study, who participate in interventions designed to promote the understanding of cultural identity as multifaceted, supporting them in the harmonization process of their various identities (Umaña-Taylor & Douglass, 2017). Fourth, the focus on Italian youth attending specific types of schools (i.e., technical and vocational) and living in a geographical area characterized by economic wealth, high population density, and a high proportion of immigrant-origin citizens (ISTAT, 2023) limits the generalizability of results. Further research is needed to replicate the study across socio-cultural and geographical contexts with different demographics and migration patterns, including other Italian regions as well as other European countries.

Despite the abovementioned limitations, our findings have several implications for diverse youth living in multicultural societies such as Italy. In the educational/school setting, interventions aiming to promote cultural identity resolution through the provision of protected spaces in which students can reflect upon and gain further awareness of their relationship with their own and others’ cultures may be beneficial for both minoritized and majority youth in terms of psychological well-being and overall school climate. Relatedly, teachers may benefit from an ad-hoc training concerning these topics to effectively support their students. In the family context, given the positive role of family ethnic socialization, raising awareness among parents and caregivers on the importance of shared conversations and appreciation/celebration of their heritage culture could further stimulate adolescents’ resolution process, especially among Italian majority youth who are generally less aware of this identity domain and therefore may benefit from increased exposure to cultural socialization. Recent contributions have highlighted that Italian youth are stuck in a dichotomy between disowning their national heritage or, on the contrary, embracing nationalist views and rejecting diversity (Save the Children, 2022). These cultural socialization processes might facilitate the harmonization of multiple cultural identities, lead to an increased sense of belonging, and foster the understanding of others who are going through the same process, thus promoting respect, empathy, and a sense of community among youth. Emphasizing openness, flexibility, and tolerance is equally important to prevent nationalist tendencies and the perpetuation of an assimilationist ideology that often characterizes countries with a recent history of immigration.

## Conclusion

Theory and empirical evidence suggest that exploring and gaining a sense of clarity regarding one’s own ethnic-racial/cultural identity is a pivotal developmental competence, with positive effects on short- and long-term adjustment. Based on this premise, the *Identity Project* intervention was designed to stimulate these processes in adolescents of any cultural background within the school setting, demonstrating efficacy in the U.S. context and, more recently, in European countries such as Italy. However, knowledge concerning specific patterns of change in exploration and resolution over time and how these may be linked to youth psychosocial outcomes is still lacking, especially among students who have participated in a school-based intervention designed to promote such processes. Using a person-centered approach, the current study addressed this gap by conducting latent profile analysis on individual process components of cultural identity (i.e., exploration and resolution) assessed four times over a school year. No subgroups of adolescents were found for exploration, whereas resolution profiles were heterogeneous, reflecting both stability and change (i.e., increase), and differed based on immigrant background as well as levels of family ethnic socialization. Overall, students in the increase profiles exhibited better psychosocial outcomes relative to their peers in the stability profiles. These findings lend support to the notion that resolution is an introspective process that differs among individuals, with personal and contextual factors influencing how this process develops. Youth who showed an increase in their sense of clarity of what it means to belong to a specific cultural group for their global self also exhibited better adjustment in the long run. From a developmental perspective, the results of this study emphasize the importance of providing youth of any cultural background with the opportunity to explore and better understand their origin(s) to promote their psychosocial well-being, although differences may exist in how individuals respond to such stimulation based on a multiplicity of factors. From an applied perspective, considering the positive role of family ethnic socialization and the different ways in which cultural identity processes developed among adolescents who participated in the Italian *Identity Project*, involving families and implementing booster sessions to keep students engaged with resolution – particularly in the months after the conclusion of the program – may represent useful strategies to maximize its efficacy.

## Supplementary Information

Online Supplementary Material

## Data Availability

The data from the current study are available in the Open Science Framework (OSF) at the following link: https://osf.io/maf4q/?view_only=d71fd907bb3c4dbe804b926b7cfa1c08.

## Footnotes

1 In logistic regression analysis, the 11 participants (6%) who identified themselves as non-binary were removed to ensure a more reliable and robust estimate of gender effects.

2 To account for the interrelatedness of exploration and resolution processes (Erikson, 1968), the same regression models were rerun including exploration at T1 and its interaction with profile membership. No significant main or interactive effects on any of the six psychosocial outcome variables emerged (see Appendix A4, Online Supplementary Material). Hence, in the paper were reported only the results of the regression models without including exploration.

## References

[CR1] Abdullahi, A. K., Syed, M., Juang, L. P., Berne, S., Hwang, C. P., & Frisén, A. (2024). *The “Identity Project” intervention in Sweden: Changes in adolescent ethnic identity process and content* [Manuscript submitted for publication]. Department of Psychology, University of Gothenburg, Sweden.

[CR2] Bañales, J., Hoffman, A. J., Rivas-Drake, D., & Jagers, R. J. (2020). The development of ethnic-racial identity process and its relation to civic beliefs among Latinx and Black American adolescents. *Journal of Youth and Adolescence*, *49*(12), 2495–2508. 10.1007/s10964-020-01254-6. 32468392 10.1007/s10964-020-01254-6

[CR3] Boyce, W., Torsheim, T., Currie, C., & Zambon, A. (2006). The Family Affluence Scale as a measure of national wealth: validation of an adolescent self-report measure. *Social Indicators Research*, *78*, 473–487. 10.1007/s11205-005-1607-6.

[CR4] Bradley, K. L., Bagnell, A. L., & Brannen, C. L. (2010). Factorial validity of the Center for Epidemiological Studies Depression 10 in adolescents. *Issues in Mental Health Nursing*, *31*(6), 408–412. 10.3109/01612840903484105. 20450343 10.3109/01612840903484105

[CR5] Brittian, A. S., Kim, S. Y., Armenta, B. E., Lee, R. M., Umaña-Taylor, A. J., Schwartz, S. J., & Hudson, M. L. (2015). Do dimensions of ethnic identity mediate the association between perceived ethnic group discrimination and depressive symptoms? *Cultural Diversity and Ethnic Minority Psychology*, *21*(1), 41–53. 10.1037/a0037531. 25090147 10.1037/a0037531 PMC7872098

[CR6] Brubaker, R. (2009). Ethnicity, race, and nationalism. *Annual Review of Sociology*, *35*(1), 21–42. 10.1146/annurev-soc-070308-115916.

[CR7] Cavdar, D., McKeown, S., & Rose, J. (2021). Mental health outcomes of ethnic identity and acculturation among British‐born children of immigrants from Turkey. *New Directions for Child and Adolescent Development*, *176*, 141–161. 10.1002/cad.20402. 10.1002/cad.20402 33683825

[CR8] Ceccon, C., Schachner, M. K., Lionetti, F., Pastore, M., Umaña‐Taylor, A. J., & Moscardino, U. (2023). Efficacy of a cultural adaptation of the Identity Project intervention among adolescents attending multiethnic classrooms in Italy: A randomized controlled trial. *Child Development*, *94*(5), 1162–1180. 10.1111/cdev.13944. 37195803 10.1111/cdev.13944

[CR9] Ceccon, C., Schachner, M. K., Umaña-Taylor, A. J., & Moscardino, U. (2024). Promoting adolescents’ cultural identity development: A pilot study of the identity project intervention in Italy. *Cultural Diversity and Ethnic Minority Psychology*. Advance online publication. 10.1037/cdp0000643. 10.1037/cdp0000643 38358649

[CR10] Chavous, T. M., Richardson, B. L., Webb, F. R., Fonseca-Bolorin, G., & Leath, S. (2018). Shifting contexts and shifting identities: Campus race-related experiences, racial identity and academic motivation among Black students during the transition to college. *Race and Social Problems*, *10*, 1–18. 10.1007/s12552-017-9218-9.

[CR11] Cohen, J. (1969). *Statistical power analysis for the behavioural sciences*. New York: Academic Press.

[CR12] Constante, K., Cross, F. L., Medina, M., & Rivas-Drake, D. (2020). Ethnic socialization, family cohesion, and ethnic identity development over time among Latinx adolescents. *Journal of Youth and Adolescence*, *49*, 895–906. 10.1007/s10964-019-01139-3. 31587174 10.1007/s10964-019-01139-3

[CR13] Currie, C., Molcho, M., Boyce, W., Holstein, B., Torsheim, T., & Richter, M. (2008). Researching health inequalities in adolescents: the development of the Health Behaviour in School-Aged Children (HBSC) family affluence scale. *Social Science & Medicine*, *66*, 1429–1436. 10.1016/j.socscimed.2007.11.024. 18179852 10.1016/j.socscimed.2007.11.024

[CR14] Dimitrova, R., Hatano, K., Sugimura, K., & Ferrer-Wreder, L. (2018). The Erikson psychosocial stage inventory in adolescent samples. *European Journal of Psychological Assessment*, *35*, 680–684. 10.1027/1015-5759/a000456.

[CR15] Dixon, T., Hawkins, S., Heijbroek, L., Juan-Torres, M., & Demoures, F.-X. (2018). *Un’Italia frammentata: atteggiamenti verso identità nazionale, immigrazione e rifugiati in Italia* [A fragmented Italy: attitudes toward national identity, immigration and refugees in Italy]. More in Common. https://www.ipsos.com/sites/default/files/ct/publication/documents/2018-08/italyitfinal_digital.pdf.

[CR16] Douglass, S., & Umaña-Taylor, A. J. (2015). Development of ethnic–racial identity among Latino adolescents and the role of family. *Journal of Applied Developmental Psychology*, *41*, 90–98. 10.1016/j.appdev.2015.09.002. 10.1037/dev0000141 27709995

[CR17] El-Tayeb, F. (2014). European others. In *Remixing Europe: Unveiling the imagery of migrants in European media*, Doc Next Network, (pp. 76–81). European Cultural Foundation.

[CR18] Erentaitė, R., Lannegrand-Willems, L., Negru-Subtirica, O., Vosylis, R., Sondaitė, J., & Raižienė, S. (2018). Identity development among ethnic minority youth: Integrating findings from studies in Europe. *European Psychologist*, *23*(4), 324 10.1027/1016-9040/a000338.

[CR19] Erikson, E. H. (1968). *Identity: Youth and crisis*. New York, NY: Norton.

[CR20] Funder, D. C., & Ozer, D. J. (2019). Evaluating effect size in psychological research: Sense and nonsense. *Advances in Methods and Practices in Psychological Science*, *2*(2), 156–168. 10.1177/251524591984.

[CR21] Garson, G. D. (2015). *Missing Values Analysis and Data Imputation*. Asheboro, NC: Statistical Associates Publishers.

[CR22] Gartner, M., Kiang, L., & Supple, A. (2014). Prospective links between ethnic socialization, ethnic and American identity, and well-being among Asian-American adolescents. *Journal of Youth and Adolescence*, *43*, 1715–1727. 10.1007/s10964-013-0044-0. 24162183 10.1007/s10964-013-0044-0

[CR23] Giofrè, D., Toffalini, E., Provazza, S., Calcagnì, A., Altoè, G., & Roberts, D. J. (2019). Are children with developmental dyslexia all the same? A cluster analysis with more than 300 cases. *Dyslexia*, *25*(3), 284–295. 10.1002/dys.1629. 31332875 10.1002/dys.1629 PMC6771784

[CR24] Gonzales-Backen, M. A., Bámaca-Colbert, M. Y., & Allen, K. (2016). Ethnic identity trajectories among Mexican-origin girls during early and middle adolescence: Predicting future psychosocial adjustment. *Developmental Psychology*, *52*(5), 790–797. 10.1037/a0040193. 26986228 10.1037/a0040193

[CR25] Harlap, Y., & Riese, H. (2022). We don’t throw stones, we throw flowers”: race discourse and race evasiveness in the Norwegian university classroom. *Ethnic and Racial Studies*, *45*(7), 1218–1238. 10.1080/01419870.2021.1904146.

[CR26] Hölscher, S. I. E., Schachner, M. K., Juang, L. P., & Altoè, G. (2024). *Promoting Adolescents’ Heritage Cultural Identity Formation in the Context of the Identity Project in Germany: The Role of Autonomy and Relatedness Basic Needs Satisfaction* [Manuscript submitted for publication]. Institute for Pedagogy, Martin Luther University of Halle-Wittenberg, Halle, Germany.

[CR27] Huang, C. Y., & Stormshak, E. A. (2011). A longitudinal examination of early adolescence ethnic identity trajectories. *Cultural Diversity and Ethnic Minority Psychology*, *17*(3), 261–270. 10.1037/a0023882. 21787058 10.1037/a0023882 PMC3144497

[CR28] Hughes, D., Rodriguez, J., Smith, E. P., Johnson, D. J., Stevenson, H. C., & Spicer, P. (2006). Parents’ ethnic-racial socialization practices: a review of research and directions for future study. *Developmental Psychology*, *42*(5), 747–770. 10.1037/0012-1649.42.5.747. 16953684 10.1037/0012-1649.42.5.747

[CR29] Huguley, J. P., Wang, M. T., Vasquez, A. C., & Guo, J. (2019). Parental ethnic–racial socialization practices and the construction of children of color’s ethnic–racial identity: A research synthesis and meta-analysis. *Psychological Bulletin*, *145*(5), 437–458. 10.1037/bul0000187. 30896188 10.1037/bul0000187

[CR30] ISTAT (2023). *Rapporto annuale 2023. La situazione del Paese* [Annual Report 2023. The situation of the country]. https://www.istat.it/storage/rapporto-annuale/2023/Rapporto-Annuale-2023.pdf.

[CR31] Juang, L. P., Schachner, M. K., Pevec, S., & Moffitt, U. (2020). The Identity Project intervention in Germany: Creating a climate for reflection, connection, and adolescent identity development. New Directions for Child and Adolescent Development, 2020, 6565–82. 10.1002/cad.20379. 10.1002/cad.20379 33108699

[CR32] Juang, L. P., Moffitt, U., Schachner, M. K., & Pevec, S. (2021). Understanding ethnic-racial identity in a context where “race” is taboo. *Identity*, *21*(3), 185–199. 10.1080/15283488.2021.1932901.

[CR33] Juang, L. P., Umaña-Taylor, A. J., Schachner, M. K., Frisén, A., Hwang, C. P., Moscardino, U., Motti-Stefanidi, M., Oppedal, B., Pavlopoulos, V., Abdullahi, A. K., Barahona, R., Berne, S., Ceccon, C., Gharaei, N., Moffitt, U., Ntalachanis, A., Pevec, S., Sandberg, D. J., Zacharia, A., & Syed, M. (2023). Ethnic-racial identity in Europe: Adapting the identity project intervention in five countries. *European Journal of Developmental Psychology*, *20*(6), 978-1006. 10.1080/17405629.2022.2131520.

[CR34] Jugert, P., Kaiser, M. J., Ialuna, F., & Civitillo, S. (2022). Researching race‐ethnicity in race‐mute Europe. *Infant and Child Development*, *31*(1), e2260 10.1002/icd.2260.

[CR35] Jugert, P., Pink, S., Fleischmann, F., & Leszczensky, L. (2020). Changes in Turkish-and resettler-origin adolescents’ acculturation profiles of identification: A three-year longitudinal study from Germany. *Journal of Youth and Adolescence*, *49*, 2476–2494. 10.1007/s10964-020-01250-w. 32405993 10.1007/s10964-020-01250-w PMC7585569

[CR36] Karataş, S., Crocetti, E., Schwartz, S. J., & Rubini, M. (2023). Developmental trajectories of ethnic and national identities in adolescents from migrant families: The role of social identification with family and classmates. *European Journal of Personality*, *37*(6), 705–722. 10.1177/08902070221149602.

[CR37] Lenth (2023). *emmeans: Estimated Marginal Means, aka Least-Squares Means*. R package version 1.8.4-1. https://CRAN.R-project.org/package=emmeans.

[CR38] Li, J. B., Delvecchio, E., Di Riso, D., Salcuni, S., & Mazzeschi, C. (2015). Self-esteem and its association with depression among Chinese, Italian, and Costa Rican adolescents: A cross-cultural study. *Personality and Individual Differences*, *82*, 20–25. 10.1016/j.paid.2015.02.036.

[CR39] Marcia, J. E. (1980). Identity in adolescence. In J. Adelson (Ed.), *Handbook of adolescent psychology* (pp. 159–187). New York, NY: Wiley.

[CR40] Meca, A., Cruz, B., Veniegas, T. K., Allison, K. K., Santibanez, L., & Gonzales-Backen, M. A. (2023). Cultural identity configurations: A latent profile analysis of ethnic/racial and US identity process and content. *Journal of Youth and Adolescence*, *52*(1), 105–121. 10.1007/s10964-022-01690-6. 36242697 10.1007/s10964-022-01690-6

[CR41] Mesurado, B., Guerra, P., De Sanctis, F., & Rodriguez, L. M. (2019). Validation of the Spanish version of the Prosocial Behavior toward Different Targets Scale. *International Social Work*, *65*(1), 175–186. 10.1177/0020872819858738.

[CR42] Miller-Cotto, D., & Byrnes, J. P. (2016). Ethnic/racial identity and academic achievement: A meta-analytic review. *Developmental Review*, *41*, 51–70. 10.1016/j.dr.2016.06.003.

[CR43] Moffit, U., & Juang, L. P. (2019). ‘We don’t do that in Germany!’A critical race theory examination of Turkish heritage young adult’s school experiences. *Ethnicities*, *19*(5), 830–857.

[CR44] Moffitt, U., Juang, L. P., & Syed, M. (2020). Intersectionality and youth identity development research in Europe. *Frontiers in Psychology*, *11*, 78 10.3389/fpsyg.2020.00078. 32082225 10.3389/fpsyg.2020.00078 PMC7005132

[CR45] Padilla‐Walker, L. M., Carlo, G., & Memmott‐Elison, M. K. (2018). Longitudinal change in adolescents’ prosocial behavior toward strangers, friends, and family. *Journal of Research on Adolescence*, *28*(3), 698–710. 10.1111/jora.12362. 29144027 10.1111/jora.12362

[CR46] Peterson, C., & Seligman, M. E. P. (2004). *Character strengths and virtues: A handbook and classification*. Washington, DC: American Psychological Association. 10.1176/appi.ajp.162.4.820-a.

[CR47] Phinney, J. S. (1989). Stages of ethnic identity development in minority group adolescents. *The Journal of Early Adolescence*, *9*, 34–49.

[CR48] Phinney, J. S. (1992). The Multigroup Ethnic Identity Measure: A new scale for use with diverse groups. *Journal of Adolescent Research*, *7*, 156–176. 10.1177/074355489272003.

[CR49] Phinney, J. S. (1993). A three-stage model of ethnic identity development in adolescence. In M. E. P. Bernal & G. P. Knight (Eds.), *Ethnic identity: Formation and transmission among Hispanics and other minorities* (pp. 61–79). New York: State University of New York Press.

[CR50] Phinney, J. S., Jacoby, B., & Silva, C. (2007). Positive intergroup attitudes: The role of ethnic identity. *International Journal of Behavioral Development*, *31*(5), 478–490. 10.1177/0165025407081466.

[CR51] Prezza, M., Trombaccia, F., & Armento, L. (1997). The rosenberg self-esteem scale: Italian translation and validation. *Bollettino di Psicologia Applicata*, *223*, 35–44.

[CR52] Radloff, L. (1977). The CES-D Scale A self-report depression scale for research in the general population. *Applied Psychological Measurement*, *7*, 385–401. 10.1177/014662167700100306.

[CR53] Raftery, A. E. (1995). Bayesian model selection in social research. *Sociological Methodology*, *25*, 111–163.

[CR54] R Core Team (2018). *R: a language and environment for statistical computing*. *R* Foundation for Statistical Computing: Vienna, Austria. https://www.R-project.org.

[CR55] Rosenberg, M. (1979). *Conceiving the self*. New York, NY: Basic Books.

[CR56] Rosenthal, D. A., Gurney, R. M., & Moore, S. M. (1981). From trust on intimacy: A new inventory examining Erikson’s stages of psychosocial development. *Journal of Youth and Adolescence*, *10*(6), 525–537. 10.1007/BF02087944. 24310543 10.1007/BF02087944

[CR57] Sandberg, D. J., Frisén, A., Juang, L. P., Hwang, C. P., & Syed, M. (2024). *Attitude change and the Identity Project: Longitudinal assessments of outgroup- and diversity attitudes among adolescents in Sweden* [Manuscript submitted for publication]. Department of Psychology, University of Gothenburg, Sweden.

[CR58] Save the Children (2022). *IMMERSE - Integration Mapping of Refugee and Migrant children in Schools and other Experiential environments in Europe. Un nuovo sguardo attraverso la ricerca qualitativa* [A new look through qualitative research]. https://s3.savethechildren.it/public/files/uploads/pubblicazioni/immerse-un-nuovo-sguardo-attraverso-la-ricerca-qualitativa_0.pdf.

[CR59] Schachner, M. K., Hölscher, S. I. E., Moscardino, U., Ceccon, C., Juang, L., & Pastore, M. (2024). *The dynamic interplay of the Identity Project intervention with classroom cultural diversity climate in Italian and German schools* [Manuscript submitted for publication]. Institute for Pedagogy, Martin Luther University of Halle-Wittenberg, Halle, Germany.

[CR60] Schachner, M. K., Juang, L., Moffitt, U., & van de Vijver, F. J. (2018). Schools as acculturative and developmental contexts for youth of immigrant and refugee background. *European Psychologist*, *23*, 44–56. 10.1027/1016-9040/a000312.

[CR61] Schachner, M. K., Noack, P., Van de Vijver, F. J., & Eckstein, K. (2016). Cultural diversity climate and psychological adjustment at school—Equality and inclusion versus cultural pluralism. *Child Development*, *87*, 1175–1191. 10.1111/cdev.12536. 27091829 10.1111/cdev.12536

[CR62] Schotte, K., Stanat, P., & Edele, A. (2018). Is integration always most adaptive? The role of cultural identity in academic achievement and in psychological adaptation of immigrant students in Germany. *Journal of Youth and Adolescence*, *47*(1), 16–37. 10.1007/s10964-017-0737-x. 28913774 10.1007/s10964-017-0737-x

[CR63] Schwarzenthal, M. (2022). Researching intercultural competence and critical consciousness among adolescents growing up in societies of immigration. *International Journal of Intercultural Relations*, *91*, 311–317. 10.1016/j.ijintrel.2022.04.011.

[CR64] Scrucca, L., Fop, M., Murphy, T. B., & Raftery, A. E. (2016). mclust 5: clustering, classification and density estimation using Gaussian finite mixture models. *The R Journal*, *8*(1), 289–317. 27818791 PMC5096736

[CR65] Skinner, E. A., Kindermann, T. A., & Furrer, C. J. (2008). A motivational perspective on engagement and disaffection: Conceptualization and assessment of children’s behavioral and emotional participation in academic activities in the classroom. *Educational and Psychological Measurement*, *69*(3), 493–525. 10.1177/0013164408323233.

[CR66] Sladek, M. R., Umaña-Taylor, A. J., McDermott, E. R., Rivas-Drake, D., & Martinez-Fuentes, S. (2020). Testing invariance of ethnic-racial discrimination and identity measures for adolescents across ethnic-racial groups and contexts. *Psychological Assessment*, *32*, 509–526. 10.1037/pas0000805. 32091231 10.1037/pas0000805

[CR67] Sladek, M. R., Umaña-Taylor, A. J., Wantchekon, K. A., McDermott, E. R., & Updegraff, K. A. (2021). Contextual moderators of a school-based ethnic-racial identity intervention: The roles of family ethnic socialization and ethnic-racial background. *Prevention Science*, 1–8. 10.1007/s11121-020-01166-8. 10.1007/s11121-020-01166-8 32996017

[CR68] Smokowski, P. R., Evans, C. B., Cotter, K. L., & Webber, K. C. (2014). Ethnic identity and mental health in American Indian youth: Examining mediation pathways through self-esteem, and future optimism. *Journal of Youth and Adolescence*, *43*, 343–355. 10.1007/s10964-013-9992-7. 23929530 10.1007/s10964-013-9992-7

[CR69] Spiegler, O., Wölfer, R., & Hewstone, M. (2019). Dual identity development and adjustment in Muslim minority adolescents. *Journal of Youth and Adolescence*, *48*, 1924–1937. 10.1007/s10964-019-01117-9. 31520235 10.1007/s10964-019-01117-9 PMC6813286

[CR70] Streit, C., Carlo, G., & Killoren, S. E. (2020). Ethnic socialization, identity, and values associated with US Latino/a young adults’ prosocial behaviors. *Cultural Diversity and Ethnic Minority Psychology*, *26*(1), 102–111. 10.1037/cdp0000280. 30920248 10.1037/cdp0000280

[CR71] Syed, M., Juang, L. P., & Svensson, Y. (2018). Toward a new understanding of ethnic‐racial settings for ethnic‐racial identity development. *Journal of Research on Adolescence*, *28*(2), 262–276. 10.1111/jora.12387. 29570904 10.1111/jora.12387

[CR72] Turner, J. C., Hogg, M. A., Oakes, P. J., Reicher, S. D., & Wetherell, M. S. (1987). *Rediscovering the social group: A self-categorization theory*. Oxford, UK: Blackwell.

[CR73] Umaña-Taylor, A. J. (2016). Ethnic-racial identity: Conceptualization, development, and associations with youth adjustment. In L. Balter & C. S. Tamis-LeMonda (Eds.), *Child psychology: A handbook of contemporary issues* (3rd ed., pp. 305–327). New York, NY: Taylor & Francis.

[CR74] Umaña-Taylor, A. J., & Douglass, S. (2017). Developing an ethnic-racial identity intervention from a developmental perspective: Process, content, and implementation. In N. J. Cabrera & B. Leyendecker (Eds.), *Handbook of positive development of minority children and youth* (pp. 437–453). Cham, Switzerland: Springer.

[CR75] Umaña‐Taylor, A. J., Douglass, S., Updegraff, K. A., & Marsiglia, F. F. (2018a). A small‐scale randomized efficacy trial of the Identity Project: Promoting adolescents’ ethnic–racial identity exploration and resolution. *Child Development*, *89*, 862–870. 10.1111/cdev.12755. 28321839 10.1111/cdev.12755

[CR76] Umaña‐Taylor, A. J., & Hill, N. E. (2020). Ethnic–racial socialization in the family: A decade’s advance on precursors and outcomes. *Journal of Marriage and Family*, *82*(1), 244–271. 10.1111/jomf.12622.

[CR77] Umaña-Taylor, A. J., Kornienko, O., Bayless, S. D., & Updegraff, K. A. (2018b). A universal intervention program increases ethnic-racial identity exploration and resolution to predict adolescent psychosocial functioning one year later. *Journal of Youth and Adolescence*, *47*, 1–15. 10.1007/s10964-017-0766-5. 29030792 10.1007/s10964-017-0766-5

[CR78] Umaña‐Taylor, A. J., Quintana, S. M., Lee, R. M., Cross, Jr, W. E., Rivas‐Drake, D., Schwartz, S. J., Syed, M., Yip, T., Seaton, E., & Ethnic and Racial Identity in the 21st Century Study Group. (2014). Ethnic and racial identity during adolescence and into young adulthood: An integrated conceptualization. *Child Development*, *85*(1), 21–39. 10.1111/cdev.12196. 24490890 10.1111/cdev.12196 PMC6673642

[CR79] Umaña-Taylor, A. J., & Rivas-Drake, D. (2021). Ethnic-racial identity and adolescents’ positive development in the context of ethnic-racial marginalization: Unpacking risk and resilience. *Human Development*, *65*, 293–310. 10.1159/000519631.

[CR80] Umaña-Taylor, A. J., Yazedjian, A., & Bámaca-Gómez, M. (2004). Developing the ethnic identity scale using Eriksonian and social identity perspectives. *Identity: An International Journal of Theory and Research*, *4*, 9–38. 10.1207/S1532706XID0401_2.

[CR81] Umaña-Taylor, A. J., Zeiders, K. H., & Updegraff, K. A. (2013). Family ethnic socialization and ethnic identity: A family-driven, youth-driven, or reciprocal process? *Journal of Family Psychology*, *27*(1), 137–146. https://psycnet.apa.org/record/2013-05310-014. 23421841 10.1037/a0031105 PMC3652612

[CR82] van der Gaag, M. A. (2023). A person‐centered approach in developmental science: Why this is the future and how to get there. *Infant and Child Development*, *32*(6), e2478 10.1002/icd.2478.

[CR83] Wantchekon, K. A., & Umaña-Taylor, A. J. (2021). Relating profiles of ethnic–racial identity process and content to the academic and psychological adjustment of Black and Latinx adolescents. *Journal of Youth and Adolescence*, *50*(7), 1333–1352. 10.1007/s10964-021-01451-x. 34085185 10.1007/s10964-021-01451-x

[CR84] Yip, T., Wang, Y., Mootoo, C., & Mirpuri, S. (2019). Moderating the association between discrimination and adjustment: A meta-analysis of ethnic/racial identity. *Developmental Psychology*, *55*(6), 1274–1298. 10.1037/dev0000708. 30907605 10.1037/dev0000708 PMC6557142

