# Supplementary material for: Longitudinal Profiles of Cultural Identity Processes and Associations with Psychosocial Outcomes Among Adolescents Participating in the Identity Project in Italy
Source: J Youth Adolesc. 2024 May 29;53(11):2443–59. doi: 10.1007/s10964-024-02022-6 (PMC11466995; doi:10.1007/s10964-024-02022-6)
Supplement: Supplementary file 1 — Online Supplementary Material [file 10964_2024_2022_MOESM1_ESM.docx]

**Longitudinal Profiles of Cultural Identity Processes and Associations with Psychosocial Outcomes Among Adolescents Participating in the *Identity Project* in Italy**

**Online Supplementary Material**

**(Appendix A)**

**Table of contents**

**A1** School demographic composition and descriptives of study variables at baseline...........................1

**A2** Multiple linear regression model with family ethnic socialization as dependent variable………...2

**A3** Multiple linear regression models with psychosocial outcomes as dependent variables, controlling for immigrant background, age, gender, and school..............................................................................3

**A4** Multiple linear regression models with psychosocial outcomes as dependent variables, including main and interactive effects of cultural identity exploration at T1……………...……………………..7

**A1 School demographic composition and descriptives of study variables at baseline**

| Variable | *School 1* | *School 2* | *School 3* |
| --- | --- | --- | --- |
|  | *M (SD)* | *M (SD)* | *M (SD)* |
| Immigrant background (% with immigrant background) | 22 | 40 | 21 |
| Socioeconomic status | 6.69 (1.56) | 6.40 (1.93) | 6.25 (1.72) |
| Age | 14.84 (0.59) | 15.05 (0.55) | 15.08 (0.67) |
| Gender (% female) | 41 | 75 | 63 |
| Cultural identity exploration (T0) | 2.51 (0.59) | 2.61 (0.57) | 2.71 (0.53) |
| Cultural identity resolution (T0) | 2.74 (0.58) | 2.87 (0.65) | 2.79 (0.64) |
| Global identity cohesion (T0) | 3.46 (0.71) | 3.14 (0.58) | 3.14 (0.61) |
| Self-esteem (T0) | 28.64 (7.36) | 23.95 (6.13) | 24.43 (6.20) |
| Depressive symptoms (T0) | 11.96 (6.42) | 15.25 (5.72) | 15.25 (6.31) |
| Academic engagement (T0) | 1.81 (0.55) | 2.33 (0.59) | 2.61 (0.39) |
| Other-group orientation (T0) | 3.36 (0.47) | 3.41 (0.44) | 3.28 (0.47) |
| Prosocial behavior (T0) | 3.90 (0.58) | 3.87 (0.66) | 3.87 (0.57) |

*Note*. Immigrant background was coded as 0 = without immigrant background (i.e., born in Italy from Italian-born parents) and 1 = with immigrant background (i.e., born in Italy or abroad from at least one parent born abroad). Gender was coded as 1 = male and 2 = female. Socioeconomic status ranges from 0 (lowest affluence) to 9 (highest affluence): 0-2 = low affluence, 3-5 = medium affluence, 6-9 = high affluence.

**A2 Multiple linear regression model with family ethnic socialization as dependent variable**

**Table A.2** Multiple linear regression model with family ethnic socialization as dependent variable.

| Variable | *B (SE)* | *Omnibus F (df)* | *η_p_*^2^ |
| --- | --- | --- | --- |
| Profile membership |  | 12.47 (3,169)*** | .18 |
| Profile 2 (stable average) | 0.57 (0.12) |  |  |
| Profile 3 (increase low-to-average) | 0.31 (0.14) |  |  |
| Profile 4 (increase high-to-higher) | 0.75 (0.14) |  |  |

*Note.* *N* = 173. Baseline category for profile membership was profile 1 (stable low). *R*^2^ = .18.

** p* < .05, ** *p* < .01, *** *p* < .001.


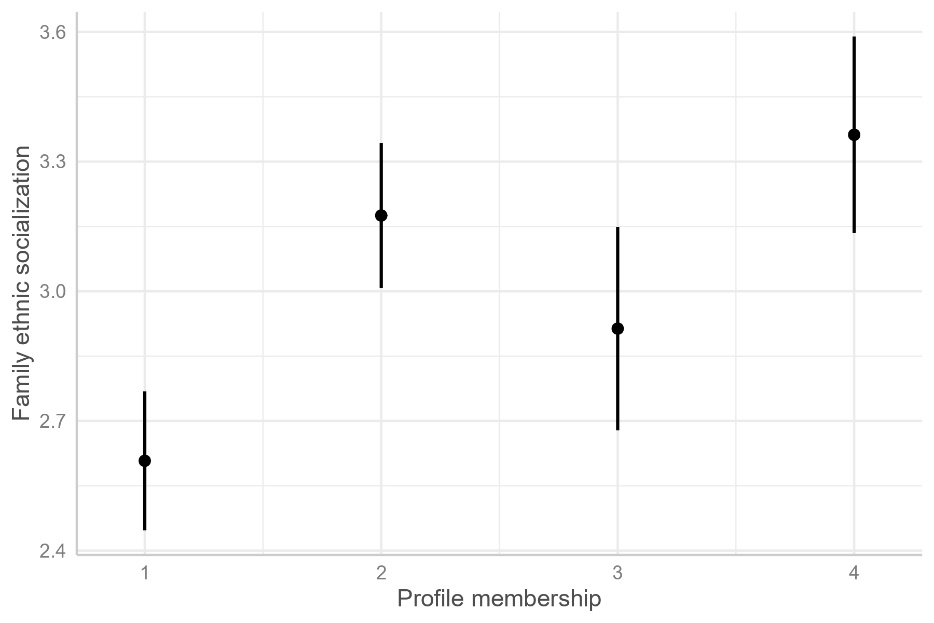
**Figure A.1** Estimated levels of family ethnic socialization at T0 across resolution profiles.

*Note*. Profile 1 “stable low” (*n* = 60), profile 2 “stable average” (*n* = 55), profile 3 “increase low-to-average” (*n* = 28), profile 4 “increase high-to-higher” (*n* = 30). Lines represent 95% confidence intervals.

**A3 Multiple linear regression models with psychosocial outcomes as dependent variables, controlling for immigrant background, age, gender, and school**

**Table A.3** Multiple linear regression model with global identity cohesion at T3 as dependent variable, controlling for immigrant background, gender, age, and school.

| Variable | *B (SE)* | *Omnibus F (df)* | *η_p_*^2^ |
| --- | --- | --- | --- |
| Global identity cohesion (T0) | 0.43 (0.05) | 82.43 (1,159)*** | .34 |
| Profile membership |  | 7.20 (3,159)*** | .12 |
| Profile 2 (stable average) | 0.05 (0.07) |  |  |
| Profile 3 (increase low-to-average) | 0.18 (0.09) |  |  |
| Profile 4 (increase high-to-higher) | 0.40 (0.09) |  |  |
| Immigrant background (immigrant) | -0.02 (0.07) | 0.06 (1,159) | < .01 |
| Gender (female) | 0.04 (0.06) | 0.43 (1,159) | < .01 |
| Age | -0.01 (0.05) | 0.06 (1,159) | < .01 |
| School |  | 0.10 (2,159) | < .01 |
| School 2 | -0.02 (0.08) |  |  |
| School 3 | 0.01 (0.07) |  |  |

*Note.* *N* = 173. Baseline category for profile membership was profile 1 (stable low). *R*^2^ = .44.

** p* < .05, ** *p* < .01, *** *p* < .001. Immigrant background was coded as 0 = without immigrant background (i.e., born in Italy from Italian-born parents) and 1 = with immigrant background (i.e., born in Italy or abroad from at least one parent born abroad). Gender was coded as 1 = male and 2 = female. Baseline category for school was school 1.

**Table A.4** Multiple linear regression model with self-esteem at T3 as dependent variable, controlling for immigrant background, gender, age, and school.

| Variable | *B (SE)* | *Omnibus F (df)* | *η_p_*^2^ |
| --- | --- | --- | --- |
| Self-esteem (T0) | 0.56 (0.05) | 103.54 (1,159)*** | .39 |
| Profile membership |  | 6.05 (3,159)*** | .10 |
| Profile 2 (stable average) | 0.36 (0.84) |  |  |
| Profile 3 (increase low-to-average) | 2.54 (1.03) |  |  |
| Profile 4 (increase high-to-higher) | 4.01 (1.09) |  |  |
| Immigrant background (immigrant) | 1.35 (0.87) | 2.40 (1,159) | .01 |
| Gender (female) | 0.09 (0.77) | 0.01 (1,159) | < .01 |
| Age | -0.37 (0.56) | 0.43 (1,159) | < .01 |
| School |  | 0.36 (2,159) | < .01 |
| School 2 | 0.83 (0.98) |  |  |
| School 3 | 0.32 (0.83) |  |  |

*Note.* *N* = 173. Baseline category for profile membership was profile 1 (stable low). *R*^2^ = .49.

** p* < .05, ** *p* < .01, *** *p* < .001. Immigrant background was coded as 0 = without immigrant background (i.e., born in Italy from Italian-born parents) and 1 = with immigrant background (i.e., born in Italy or abroad from at least one parent born abroad). Gender was coded as 1 = male and 2 = female. Baseline category for school was school 1.

**Table A.5** Multiple linear regression model with depressive symptoms at T3 as dependent variable, controlling for immigrant background, gender, age, and school.

| Variable | *B (SE)* | *Omnibus F (df)* | *η_p_*^2^ |
| --- | --- | --- | --- |
| Depressive symptoms (T0) | 0.60 (0.06) | 109.59 (1,159)*** | 0.41 |
| Profile membership |  | 3.00 (3,159)* | .05 |
| Profile 2 (stable average) | -1.24 (0.79) |  |  |
| Profile 3 (increase low-to-average) | -1.85 (0.96) |  |  |
| Profile 4 (increase high-to-higher) | -2.84 (1.02) |  |  |
| Immigrant background (immigrant) | 0.57 (0.81) | 0.50 (1,159) | < .01 |
| Gender (female) | 0.11 (0.76) | 0.02 (1,159) | < .01 |
| Age | -0.62 (0.53) | 1.40 (1,159) | .01 |
| School |  | 7.28 (2,159)*** | .08 |
| School 2 | -2.04 (0.90) |  |  |
| School 3 | -2.91 (0.77) |  |  |

*Note.* *N* = 173. Baseline category for profile membership was profile 1 (stable low). *R*^2^ = .49.

** p* < .05, ** *p* < .01, *** *p* < .001. Immigrant background was coded as 0 = without immigrant background (i.e., born in Italy from Italian-born parents) and 1 = with immigrant background (i.e., born in Italy or abroad from at least one parent born abroad). Gender was coded as 1 = male and 2 = female. Baseline category for school was school 1.

**Table A.6** Multiple linear regression model with academic engagement at T3 as dependent variable, controlling for immigrant background, gender, age, and school.

| Variable | *B (SE)* | *Omnibus F (df)* | *η_p_*^2^ |
| --- | --- | --- | --- |
| Academic engagement (T0) | 0.44 (0.07) | 38.29 (1,159)*** | .19 |
| Profile membership |  | 1.68 (3,159) | .03 |
| Profile 2 (stable average) | 0.09 (0.08) |  |  |
| Profile 3 (increase low-to-average) | 0.14 (0.10) |  |  |
| Profile 4 (increase high-to-higher) | 0.24 (0.11) |  |  |
| Immigrant background (immigrant) | 0.13 (0.09) | 2.23 (1,159) | .01 |
| Gender (female) | 0.05 (0.08) | 0.39 (1,159) | < .01 |
| Age | -0.05 (0.06) | 0.77 (1,159) | < .01 |
| School |  | 2.49 (2,159) | .03 |
| School 2 | -0.10 (0.10) |  |  |
| School 3 | -0.22 (0.10) |  |  |

*Note.* *N* = 173. Baseline category for profile membership was profile 1 (stable low). *R*^2^ = .28.

** p* < .05, ** *p* < .01, *** *p* < .001. Immigrant background was coded as 0 = without immigrant background (i.e., born in Italy from Italian-born parents) and 1 = with immigrant background (i.e., born in Italy or abroad from at least one parent born abroad). Gender was coded as 1 = male and 2 = female. Baseline category for school was school 1.

**Table A.7** Multiple linear regression model with other group orientation at T3 as dependent variable, controlling for immigrant background, gender, age, and school.

| Variable | *B (SE)* | *Omnibus F (df)* | *η_p_*^2^ |
| --- | --- | --- | --- |
| Other group orientation (T0) | 0.52 (0.08) | 40.40 (1,159)*** | 0.20 |
| Profile membership |  | 0.65 (3,159) | 0.01 |
| Profile 2 (stable average) | 0.11 (0.09) |  |  |
| Profile 3 (increase low-to-average) | 0.03 (0.11) |  |  |
| Profile 4 (increase high-to-higher) | 0.11 (0.11) |  |  |
| Immigrant background (immigrant) | 0.17 (0.09) | 3.28 (1,159) | .02 |
| Gender (female) | 0.18 (0.08) | 5.03 (1,159)* | .03 |
| Age | -0.10 (0.06) | 2.64 (1,159) | .02 |
| School |  | 2.48 (2,159) | .03 |
| School 2 | -0.23 (0.10) |  |  |
| School 3 | -0.11 (0.09) |  |  |

*Note.* *N* = 173. Baseline category for profile membership was profile 1 (stable low). *R*^2^ = .32.

** p* < .05, ** *p* < .01, *** *p* < .001. Immigrant background was coded as 0 = without immigrant background (i.e., born in Italy from Italian-born parents) and 1 = with immigrant background (i.e., born in Italy or abroad from at least one parent born abroad). Gender was coded as 1 = male and 2 = female. Baseline category for school was school 1.

**Table A.8** Multiple linear regression model with prosocial behavior at T3 as dependent variable, controlling for immigrant background, gender, age, and school.

| Variable | *B (SE)* | *Omnibus F (df)* | *η_p_*^2^ |
| --- | --- | --- | --- |
| Prosocial behavior (T0) | 0.62 (0.08) | 58.82 (1,159)*** | .27 |
| Profile membership |  | 5.45 (3,159)** | .09 |
| Profile 2 (stable average) | -0.01 (0.11) |  |  |
| Profile 3 (increase low-to-average) | 0.30 (0.14) |  |  |
| Profile 4 (increase high-to-higher) | 0.48 (0.15) |  |  |
| Immigrant background (immigrant) | -0.01 (0.12) | 0.01 (1,159) | < .01 |
| Gender (female) | 0.06 (0.10) | 0.37 (1,159) | < .01 |
| Age | -0.01 (0.08) | 0.01 (1,159) | < .01 |
| School |  | 1.67 (2,159) | .02 |
| School 2 | 0.18 (0.13) |  |  |
| School 3 | 0.19 (0.11) |  |  |

*Note.* *N* = 173. Baseline category for profile membership was profile 1 (stable low). *R*^2^ = .37.

** p* < .05, ** *p* < .01, *** *p* < .001. Immigrant background was coded as 0 = without immigrant background (i.e., born in Italy from Italian-born parents) and 1 = with immigrant background (i.e., born in Italy or abroad from at least one parent born abroad). Gender was coded as 1 = male and 2 = female. Baseline category for school was school 1.

**Figure A.2** Estimated levels of global identity cohesion [panel A], self-esteem [panel B], depressive symptoms [panel C], academic engagement [panel D], other group orientation [panel E] and prosocial behavior [panel f] at T3 across resolution profiles.


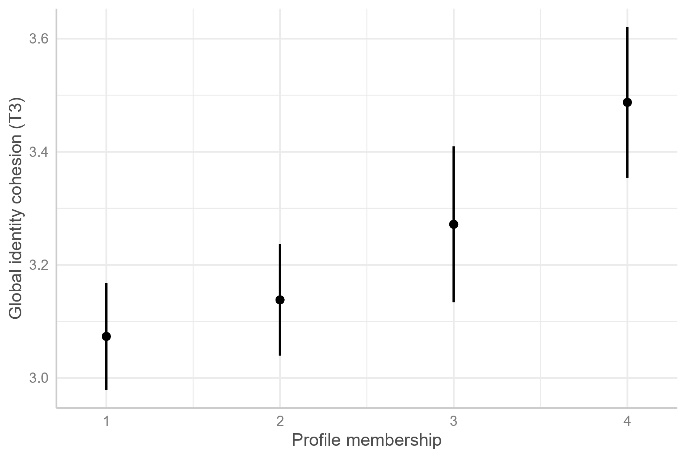

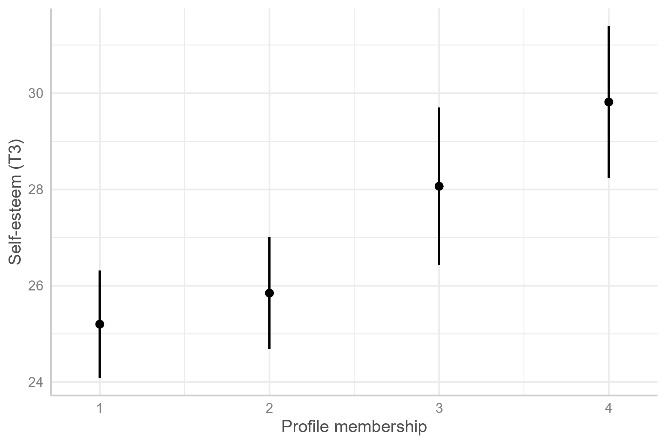
[A] [B]


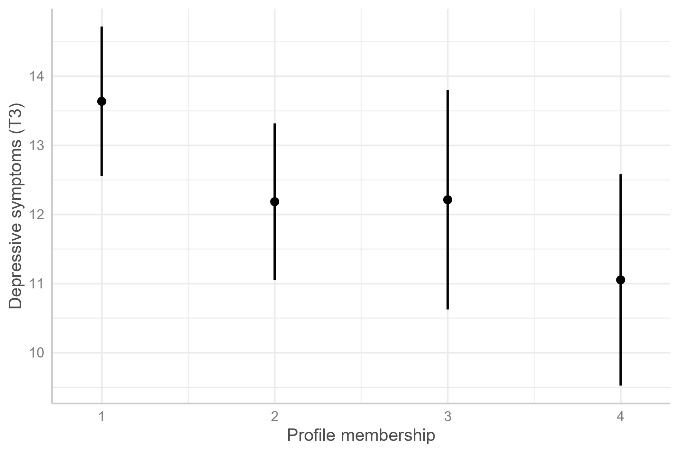

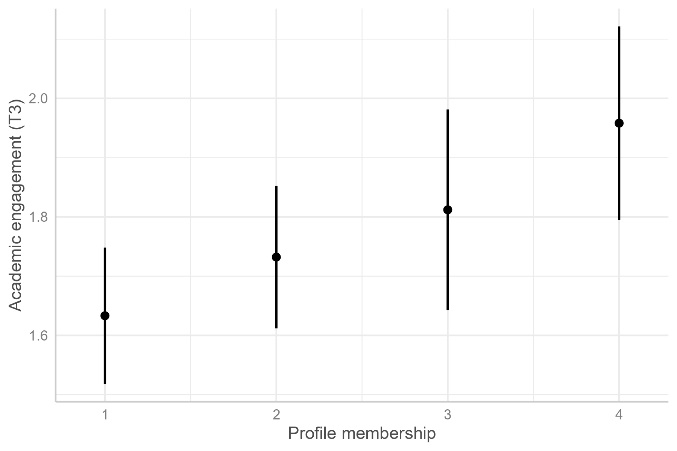
[C] [D]


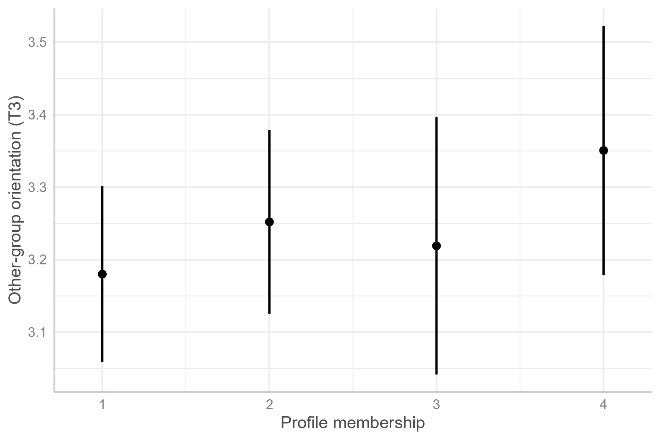

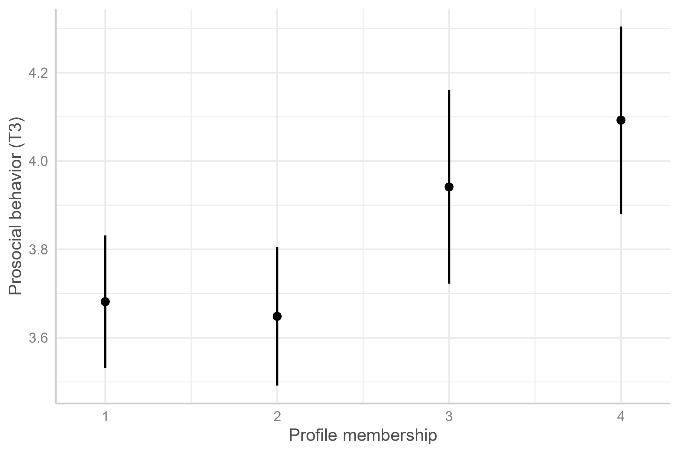
[E] [F]

*Note*. Profile 1 “stable low” (*n* = 60), profile 2 “stable average” (*n* = 55), profile 3 “increase low-to-average” (*n* = 28), profile 4 “increase high-to-higher” (*n* = 30). Lines represent 95% confidence intervals.

**A4 Multiple linear regression models with psychosocial outcomes as dependent variables, including main and interactive effects of cultural identity exploration at T1**

**Table A.9** Multiple linear regression model with global identity cohesion at T3 as dependent variable, including the main and interactive effects of cultural identity exploration at T1.

| Variable | *B (SE)* | *Omnibus F (df)* | *η_p_*^2^ |
| --- | --- | --- | --- |
| Global identity cohesion (T0) | 0.44 (.04) | 99.41 (1,164)*** | .38 |
| Profile membership |  | 8.36 (3,164)*** | .13 |
| Profile 2 (stable average) | -0.30 (0.37) |  |  |
| Profile 3 (increase low-to-average) | 0.40 (0.64) |  |  |
| Profile 4 (increase high-to-higher) | -0.17 (0.45) |  |  |
| Exploration (T1) | -0.06 (0.09) | 0.10 (1,164) | < .01 |
| Exploration x Profile membership |  | 0.85 (3,164) | .01 |
| Exploration x Profile 2 | 0.14 (0.14) |  |  |
| Exploration x Profile 3 | -0.07 (0.23) |  |  |
| Exploration x Profile 4 | 0.21 (0.16) |  |  |

*Note.* *N* = 173. Baseline category for profile membership was profile 1 (stable low). *R*^2^ = .47.

** p* < .05, ** *p* < .01, *** *p* < .001.

**Table A.10** Multiple linear regression model with self-esteem at T3 as dependent variable, including main and interactive effects of cultural identity exploration at T1.

| Variable | *B (SE)* | *Omnibus F (df)* | *η_p_*^2^ |
| --- | --- | --- | --- |
| Self-esteem (T0) | 0.56 (0.05) | 132.36 (1,164)*** | .45 |
| Profile membership |  | 9.09 (3,164)*** | .14 |
| Profile 2 (stable average) | -2.25 (4.39) |  |  |
| Profile 3 (increase low-to-average) | 1.81 (7.52) |  |  |
| Profile 4 (increase high-to-higher) | -7.48 (5.22) |  |  |
| Exploration (T1) | -1.70 (1.08) | 0.29 (1,164) | < .01 |
| Exploration x Profile membership |  | 1.94 (3,164) | .03 |
| Exploration x Profile 2 | 1.18 (1.61) |  |  |
| Exploration x Profile 3 | 0.52 (2.70) |  |  |
| Exploration x Profile 4 | 4.34 (1.83) |  |  |

*Note.* *N* = 173. Baseline category for profile membership was profile 1 (stable low). *R*^2^ = .52.

** p* < .05, ** *p* < .01, *** *p* < .001.

**Table A.11** Multiple linear regression model with depressive symptoms at T3 as dependent variable, including main and interactive effects of cultural identity exploration at T1.

| Variable | *B (SE)* | *Omnibus F (df)* | *η_p_*^2^ |
| --- | --- | --- | --- |
| Depressive symptoms (T0) | 0.55 (0.05) | 112.08 (1,164)*** | .41 |
| Profile membership |  | 3.23 (3,164)* | .06 |
| Profile 2 (stable average) | 0.93 (4.29) |  |  |
| Profile 3 (increase low-to-average) | 4.76 (7.32) |  |  |
| Profile 4 (increase high-to-higher) | -5.75 (5.10) |  |  |
| Exploration (T1) | 1.17 (1.07) | 1.71 (1,164) | .01 |
| Exploration x Profile membership |  | 0.61 (3,164) | .01 |
| Exploration x Profile 2 | -0.96 (1.57) |  |  |
| Exploration x Profile 3 | -2.31 (2.63) |  |  |
| Exploration x Profile 4 | 0.92 (1.79) |  |  |

*Note.* *N* = 173. Baseline category for profile membership was profile 1 (stable low). *R*^2^ = .46.

** p* < .05, ** *p* < .01, *** *p* < .001.

**Table A.12** Multiple linear regression model with academic engagement at T3 as dependent variable, including main and interactive effects of cultural identity exploration at T1.

| Variable | *B (SE)* | *Omnibus F (df)* | *η_p_*^2^ |
| --- | --- | --- | --- |
| Academic engagement (T0) | 0.36 (0.06) | 39.98 (1,164)*** | .20 |
| Profile membership |  | 2.64 (3,164) | .05 |
| Profile 2 (stable average) | 0.19 (0.46) |  |  |
| Profile 3 (increase low-to-average) | 0.39 (0.78) |  |  |
| Profile 4 (increase high-to-higher) | 0.07 (0.54) |  |  |
| Exploration (T1) | 0.11 (0.11) | 2.30 (1,164) | .01 |
| Exploration x Profile membership |  | 0.14 (3,164) | < .01 |
| Exploration x Profile 2 | -0.04 (0.17) |  |  |
| Exploration x Profile 3 | -0.09 (0.28) |  |  |
| Exploration x Profile 4 | 0.07 (0.19) |  |  |

*Note.* *N* = 173. Baseline category for profile membership was profile 1 (stable low). *R*^2^ = .26.

** p* < .05, ** *p* < .01, *** *p* < .001.

**Table A.13** Multiple linear regression model with other-group orientation at T3 as dependent variable, including main and interactive effects of cultural identity exploration at T1.

| Variable | *B (SE)* | *Omnibus F (df)* | *η_p_*^2^ |
| --- | --- | --- | --- |
| Other-group orientation (T0) | 0.55 (0.08) | 46.18 (1,164)*** | .22 |
| Profile membership |  | 0.45 (3,164) | .01 |
| Profile 2 (stable average) | 0.09 (0.48) |  |  |
| Profile 3 (increase low-to-average) | -0.03 (0.82) |  |  |
| Profile 4 (increase high-to-higher) | -0.42(0.57) |  |  |
| Exploration (T1) | 0.10 (0.12) | 3.25 (1,164) | .02 |
| Exploration x Profile membership |  | 0.39 (3,164) | .01 |
| Exploration x Profile 2 | -0.02 (0.18) |  |  |
| Exploration x Profile 3 | 0.01 (0.29) |  |  |
| Exploration x Profile 4 | 0.19 (0.20) |  |  |

*Note.* *N* = 173. Baseline category for profile/profile membership was profile 1 (stable low). *R*^2^ = .28.

** p* < .05, ** *p* < .01, *** *p* < .001.

**Table A.14** Multiple linear regression model with prosocial behavior at T3 as dependent variable, including main and interactive effects of cultural identity exploration at T1.

| Variable | *B (SE)* | *Omnibus F (df)* | *η_p_*^2^ |
| --- | --- | --- | --- |
| Prosocial behavior (T0) | 0.63 (0.08) | 66.21 (1,164)*** | .29 |
| Profile membership |  | 0.05 (3,164) | < .01 |
| Profile 2 (stable average) | 0.82 (0.59) |  |  |
| Profile 3 (increase low-to-average) | 2.12 (1.01) |  |  |
| Profile 4 (increase high-to-higher) | 0.22 (0.71) |  |  |
| Exploration (T1) | 0.12 (0.15) | 5.00 (1,164)* | .08 |
| Exploration x Profile membership |  | 1.91 (3,164) | .03 |
| Exploration x Profile 2 | -0.32 (0.22) |  |  |
| Exploration x Profile 3 | -0.68 (0.36) |  |  |
| Exploration x Profile 4 | 0.05 (0.25) |  |  |

*Note.* *N* = 173. Baseline category for profile/profile membership was profile 1 (stable low). *R*^2^ = .37.

** p* < .05, ** *p* < .01, *** *p* < .001.
